# Effects of Dietary Copper on Growth Performance, Antioxidant Capacity, Muscle Quality and the Expression of Copper-Transport and Muscle Development-Related Genes in Juvenile Red Swamp Crayfish (*Procambarus clarkii*)

**DOI:** 10.3390/ani16182949

**Published:** 2026-09-19

**Authors:** Feifan Xu, Xiaochuan Zheng, Yucheng Lou, Yebing Yu, Xuwen Bing, Cunxin Sun, Bo Liu, Jian Wang, Bo Liu

**Affiliations:** 1 Wuxi Fisheries College, Nanjing Agricultural University, No. 9 Shanshui East Road, Wuxi 214081, China; xufeifan@stu.njau.edu.cn (F.X.); bingxw@ffrc.cn (X.B.); suncx@ffrc.cn (C.S.); 2 Key Laboratory of Freshwater Fisheries and Germplasm Resources Utilization, Ministry of Agriculture and Rural Affairs, Freshwater Fisheries Research Center, Chinese Academy of Fishery Sciences, Wuxi 214081, China; liubo96@ffrc.cn; 3 School of Marine and Bioengineering, Yancheng Institute of Technology, Yancheng 224051, China; koikoi070803@outlook.com (Y.L.); yuyebing2005@126.com (Y.Y.); 4 Suzhou Yangcheng Lake Modern Agriculture Development Co., Ltd., No. 1 Xiangshi Road, Yangchenghu Town, Suzhou 215138, China; 18912730001@163.com

**Keywords:** Cu^2+^, *Procambarus clarkii*, growth performance, antioxidant capacity, muscle quality

## Abstract

This study assessed the influence of different dietary Cu^2+^ concentrations (1.48, 12.72, 27.71, 65.09, and 121.34 mg/kg) on the growth and physiology of juvenile crayfish (1.84 ± 0.01 g) during an 8-week feeding trial. A total of 500 crayfish were randomly allocated to the five dietary treatments, with four replicate cement ponds per treatment and 25 crayfish per tank. Growth performance was optimal in the 27.71 mg/kg group; relative to the 1.48 mg/kg group, weight gain rate (WGR) increased by 18.6% (from 749.74 ± 3.58% to 889.54 ± 9.18%), specific growth rate (SGR) by 7.3% (from 3.82 ± 0.01 to 4.10 ± 0.02%/d), and feed conversion ratio (FCR) decreased by 21.3% (from 1.69 ± 0.03 to 1.33 ± 0.02) (*p* < 0.05). Cu^2+^ deposition in muscle, hemolymph and hepatopancreas increased with dietary Cu^2+^ concentrations, accompanied by elevated transcriptional levels of copper-transporting ATPase 7 (*ATP7*) and a peak in copper transporter 1 (*Ctr1*) in the 27.71 mg/kg group. Phenoloxidase (PO) concentration and copper/zinc superoxide dismutase (Cu/Zn-SOD) activity in hemolymph peaked in the group of 27.71 mg/kg Cu^2+^, whereas malondialdehyde (MDA) concentrations reached their lowest. Excessive Cu^2+^ (121.34 mg/kg) induced oxidative stress and suppressed growth performance. 27.71 mg/kg Cu^2+^ significantly increased the hardness, gumminess and shear force of crayfish muscle compared with the other groups (*p* < 0.05). Meanwhile, this Cu^2+^ concentration increased muscle fiber diameter, an effect that was accompanied by lower myostatin (*MSTN*) and higher myosin heavy chain (*MyHC*) transcript levels in muscle. Additionally, 27.71 mg/kg Cu^2+^ significantly increased hydroxyproline (HYP) content (*p* < 0.05). Based on WGR and FCR, a segmented linear regression analysis was conducted, estimating the optimal dietary Cu^2+^ requirements for juvenile *P. clarkii* to be 28.57 and 31.50 mg/kg, respectively. These findings provide novel insights into Cu^2+^-regulated growth and muscle quality in crayfish aquaculture.

## Simple Summary

The red swamp crayfish is a globally important freshwater aquaculture species, yet producers lack evidence-based dietary Cu^2+^ guidelines, as both Cu^2+^ deficiency and excess impair animal health, growth performance and product quality. This study aimed to determine the optimal dietary Cu^2+^ level for juvenile red swamp crayfish and clarify how Cu^2+^ affects its growth, ability to resist cellular damage, and muscle quality. We conducted an 8-week feeding trial with five graded dietary Cu^2+^ concentrations. Growth performance and feed efficiency were optimised at 28.57–31.50 mg copper per kg diet. Muscle texture and composition were also modified within this range, with the highest hardness, gumminess, shear force and collagen content but the lowest crude lipid content. These findings provide a science-based dietary standard for the crayfish feed industry, supporting improved production efficiency, reduced resource waste, enhanced product quality for consumers, and more sustainable aquaculture practices.

## 1. Introduction

The red swamp crayfish, *Procambarus clarkii*, native to northern Mexico and the southern United States [1], has become one of the most important farmed freshwater crustaceans worldwide, owing to its rapid growth, early sexual maturation and tolerance of seasonal water bodies [2]. Its muscle is rich in high-quality protein and contains a favourable fatty acid profile, which underpins its popularity among consumers in many countries [3]. Sustaining this industry requires nutritionally optimised and cost-efficient formulated feeds. To date, however, nutritional research on *P. clarkii* has focused largely on macronutrients (protein, lipid and carbohydrate) [4,5,6], whereas the dietary requirements for essential trace elements, particularly at different developmental stages, remain uncharacterised. This knowledge gap constrains both the efficiency and the sustainability of its culture.

Copper (Cu) is an essential trace element for aquatic animals, supporting growth and fundamental physiological processes [7]. It acts mainly as a catalytic or structural component of cuproenzymes and Cu-dependent proteins, including hemocyanin, copper–zinc superoxide dismutase, cytochrome c oxidase, lysyl oxidase, tyrosinase and phenoloxidase [8,9], through which it participates in oxygen transport [10], antioxidant defense [11], energy production [12], collagen synthesis [13], melanogenesis [14] and immune responses [15]. Dietary Cu^2+^ can further enhance intestinal digestive enzyme activity [16], histidine absorption [17] and intestinal microbiota composition [18], thereby facilitating nutrient utilisation and growth. Both Cu^2+^ deficiency and Cu^2+^ toxicity, however, are detrimental to growth, digestive health and immune competence [7]. Reported dietary Cu^2+^ requirements range from 2 to 22.6 mg/kg in fish [19] and from 10 to 61.9 mg/kg in crustaceans [20,21], yet the requirement of juvenile *P. clarkii* remains unknown. In the present study, this requirement was estimated using standard growth indices—weight gain rate (WGR), specific growth rate (SGR) and feed conversion ratio (FCR); furthermore, to investigate the mechanisms underlying Cu^2+^ homeostasis, Cu^2+^ status was characterised by tissue Cu^2+^ accumulation and by the expression of two key Cu^2+^ transporters: copper transporter 1 (*Ctr1*), which mediates cellular Cu^2+^ uptake, and copper-transporting ATPase 7 (*ATP7*), which mediates Cu^2+^ efflux [22,23].

A further challenge in *P. clarkii* aquaculture is the inconsistency of muscle development, which frequently produces a high proportion of small individuals with underdeveloped musculature, characterised by reduced myofiber diameter and collagen deposition. This suboptimal development can impair molting success and structural support, and thereby survival and final biomass yield. Nutritional strategies that optimise muscle development and flesh quality have consequently received increasing attention [24,25], and minerals are recognised as important determinants of muscle development and product quality [26,27]. In livestock and poultry, dietary Cu^2+^ improves meat quality by promoting collagen synthesis and muscle fiber development [28,29,30,31], and Cu^2+^ has likewise been shown to positively regulate collagen deposition and myofiber growth in grass carp (*Ctenopharyngodon idella*) [32]. In *P. clarkii*, by contrast, Cu^2+^ research has concentrated on the toxicological effects of Cu^2+^ exposure on antioxidant and immune responses [13,33,34], and little is known about its nutritional role in muscle development. Our laboratory previously estimated the dietary Cu^2+^ requirement of sub-adult *P. clarkii* at 46.24–47.86 mg/kg [35]; however, the requirement of juveniles and the mechanisms by which Cu^2+^ affects muscle development remain unexplored. The present study was therefore designed to investigate the effects of graded dietary Cu^2+^ on growth performance, antioxidant capacity and muscle quality in juvenile *P. clarkii*.

To clarify how Cu^2+^ may improve muscle development, two interrelated pathways were examined: collagen deposition and myofiber development. Collagen, particularly type I, is the principal structural protein determining flesh texture, and its synthesis is regulated at both the transcriptional and the translational level—transcriptionally by the TGF-β1/Smads signaling pathway, a key regulator of collagen gene expression [36], and translationally by La ribonucleoprotein 6 (*LARP6*), an RNA-binding protein that regulates translation initiation of type I collagen mRNAs [37]. Myofiber development was assessed using myostatin (*MSTN*), a negative regulator of muscle growth [38]; myosin heavy chain (*MyHC*), a marker of myofiber type and hypertrophy [39]; and the myocyte enhancer factor-2 family (mef2a, *mef2b*), which is essential for myogenesis [40]. Because these processes are governed by nutrient sensing, key components of the mTOR pathway (*mTOR*, *S6K1* and *AKT*), which promote protein synthesis, and forkhead box O (*FOXO*), which is associated with protein degradation, were also analysed [41]. These pathways are, however, not independent of cellular Cu^2+^ handling. In crustaceans, *Ctr1*-mediated Cu^2+^ uptake and *ATP7*-mediated Cu^2+^ efflux and compartmentalisation jointly maintain Cu^2+^ homeostasis and thereby control the Cu^2+^ available for cuproenzymes—notably lysyl oxidase, which cross-links collagen and elastin, and Cu/Zn-SOD, which sustains antioxidant defence in muscle [8]. The *Ctr1*/*ATP7* system thus provides the mechanistic link between dietary Cu^2+^ supply and the collagen deposition and myofiber development pathways described above, although this remains to be experimentally verified. Muscle quality is a critical economic trait that directly influences consumer acceptance and market value [19], and inconsistent muscle texture and nutritional composition remain major constraints on profitability and consumer satisfaction in *P. clarkii* farming. These molecular responses were therefore further related to commercially relevant phenotypes using a multiparametric approach: nutritional composition (crude protein, crude lipid and collagen content, measured as hydroxyproline), textural properties (hardness, shear force and TPA parameters) and structural characteristics (myofiber diameter). Collectively, these integrated analyses bridge physiological regulation with commercially relevant phenotypic traits and provide a robust assessment of how dietary Cu^2+^ modulates muscle quality.

In the physio-biochemical assessment, hemolymph endpoints were prioritised over hepatopancreatic analyses. Although the hepatopancreas is the main organ for dietary Cu^2+^ storage and detoxification in crustaceans [42], hemolymph is a key circulatory and immune medium and an important site of Cu^2+^ transport, distribution and physiological function [43]; hemolymph endpoints therefore provide an integrated profile of the nutritional and physiological status of the animal. Total protein (TP), albumin (ALB), glucose (GLU) and triglycerides (TG) were measured as direct indicators of nutrient metabolism and energy balance [35]. Because Cu^2+^ is an essential cofactor of copper–zinc superoxide dismutase (Cu/Zn-SOD), phenoloxidase (PO) and hemocyanin (HC), the activities of these molecules reliably reflect antioxidant capacity and immune competence under different dietary Cu^2+^ regimes [44,45], while the metabolic enzymes AST and ALT and the lipid peroxidation marker MDA serve as sensitive indices of tissue integrity and oxidative stress, respectively [21]. Collectively, these biomarkers allow a direct assessment of how dietary Cu^2+^ modulates the balance among nutrient metabolism, oxidative stress and immune function.

Specifically, the objectives of this study were to (1) determine the optimal dietary Cu^2+^ requirement of juvenile *P. clarkii* based on growth performance and (2) elucidate the potential mechanisms by which Cu^2+^ influences muscle quality, with emphasis on myofiber development and collagen synthesis. The findings are expected to provide a basis for formulating balanced feeds and to support the sustainable aquaculture of this economically important species.

## 2. Materials and Methods

### 2.1. Experimental Diets

Copper was supplemented into five isonitrogenous and isolipidic diets at intended concentrations of 0, 15, 30, 60 and 120 mg Cu^2+^/kg diet (as Cu^2+^, supplied as CuSO_4_·5H_2_O, the Chinese National Standard GB/T 665-2007 [46], CuSO_4_·5H_2_O content ≥ 99.0%). The various raw ingredients were first ground and then passed through a 60-mesh sieve. The powdered ingredients were then mixed in a stepwise manner and thoroughly blended to achieve a uniform consistency. Subsequently, lipid sources and water (constituting 25% of the mixture) were added. To exclude background interference from trace minerals, the vitamin and mineral premix used in this study was custom-formulated to be copper-free. The Cu^2+^ supplement was introduced into the mixture in an aqueous solution. This mixture was then processed using a twin-screw extruder (F-26 type, Guangzhou Huagong Optical Mechanical and Electrical Technology Co., Ltd., Guangzhou, China) to produce moist pellets with a diameter of 1.5 mm. The resulting pellets were air-dried to a moisture content of approximately 10% and then stored at −20 °C until use. Following feed preparation, proximate composition and Cu^2+^ concentrations were analyzed as described in Section 2.5. The measured Cu^2+^ contents in the final diets were 1.48, 12.72, 27.71, 65.09, and 121.34 mg/kg, corresponding to the groups labeled Cu1.48, Cu12.72, Cu27.71, Cu65.09, and Cu121.34. The full formulation of the experimental diets is presented in Table 1.

### 2.2. Animals and Feeding Management

The formal feeding trial lasted 8 weeks and was carried out at the Jingjiang Experimental Base of the Freshwater Fisheries Research Center (FFRC), Chinese Academy of Fishery Sciences (CAFS). Juvenile *Procambarus clarkii* were obtained from this base. After being transported back, the crayfish were temporarily cultured in cement ponds (2 m × 1.45 m × 0.3 m, length × width × water depth) and fed a commercial diet for one week to acclimate to the farming environment. The water used in the study was local tap water (Jingjiang), which was aerated for three days before use. The commercial diet used was the Anyou 8233 formula feed, sourced from Nantong Anyou Biotechnology Group Co., Ltd. (Nantong, China). Its nutritional composition included 33% crude protein, 8% crude fiber, 5% crude fat, and 19% crude ash. The feed contained approximately 20 mg/kg Cu^2+^. Before the trial, the experimental crayfish were starved for 24 h. Then, 500 healthy juvenile crayfish with intact limbs, robust physique, and uniform size (initial body weight: 1.84 ± 0.01 g) were selected and randomly distributed into 20 cement ponds, with 25 individuals per cement pond. Each dietary treatment was randomly assigned to four replicate cement ponds. Simultaneously, 10 crayfish were randomly selected as initial whole-crayfish samples and stored in a −20 °C freezer. Prior to sampling, the animals were anesthetized with clove oil, followed by rapid chilling for euthanasia. The crayfish were fed twice daily (at 8:00 and 19:00) at a daily feeding rate equivalent to 3–10% of their total biomass for eight weeks. Following a 2 h feeding period, uneaten feed was collected via siphoning. The samples were subsequently dried in an oven at 60 °C, and the feed intake was determined. Throughout the trial, the feed amount was adjusted daily based on the remaining feed to ensure satiety. Key water quality parameters, measured weekly, remained within the following ranges: temperature 25–28 °C, pH 7.8–8.0, dissolved oxygen 8–9 mg/L, nitrite 0.01–0.05 mg/L, and ammonia below 0.05 mg/L. Each cement pond was operated as an independent static rearing unit without a recirculating filtration system. Water quality was therefore maintained by manual water exchange and periodic sludge removal. Approximately one-third of the rearing water (10 cm) was renewed every three days with fully aerated tap water from the same source. Faeces, molt residues and other settled debris were removed once a week using an electric sludge suction device, after which the water level was restored with aerated tap water. To reduce cannibalistic behavior, various types of shelters—including artificial nests and PVC tubes—were placed in each cement pond.

### 2.3. Sample Collection

Following the experimental period, a comprehensive assessment of growth indicators and feed efficiency was conducted. This evaluation was based on the total biomass and population size of crayfish in each cement pond, determined by counting and weighing the entire population. Two crayfish per cement pond were randomly selected and preserved at −20 °C for the determination of whole-body proximate composition. Prior to sampling, the animals were anesthetized with clove oil (500 μL/L) [47], followed by rapid chilling for euthanasia. Eight crayfish were randomly selected from each replicate pond for measurements of body length, total weight, and the weights of muscle and hepatopancreas (the weight of muscle refers to the weight of the abdominal muscle tissue from the shrimp tail). Hemolymph was collected from four randomly selected crayfish per cement pond. Before sampling, 2 mL sterile syringes fitted with 26G needles were rinsed with pre-cooled (4 °C) anticoagulant solution (14.7 g/L glucose, 13.2 g/L sodium citrate, 4.8 g/L citric acid). Each crayfish was held with the dorsal side up, and the needle was inserted at a 45° angle to the body surface into the pericardial sinus through the dorsal posterior cephalothorax (immediately posterior to the cervical groove and slightly lateral to the dorsal midline), to a depth of 3–5 mm. Approximately 0.6 mL of hemolymph was withdrawn per individual and immediately mixed with an equal volume (1:1, *v*/*v*) of the anticoagulant solution. The mixture was then transferred to an anticoagulant tube and centrifuged at 3000 r/min for 15 min at 4 °C to separate the hemolymph plasma from the blood cells. The resulting supernatant (plasma) was collected and stored at −20 °C for subsequent analysis. All dissection procedures were performed aseptically using stainless steel instruments. The carapace was first carefully removed to expose internal tissues, including the hepatopancreas and abdominal muscles. The hepatopancreas was carefully dissected out after removal of the carapace. It was gently separated from surrounding tissues to maintain structural integrity and prevent content leakage. For muscle sampling, the abdominal tail muscle located anterior to the tail fan was selected due to its large mass and high homogeneity. The overlying chitinous exoskeleton was carefully dissected away to obtain pure muscle samples. Fresh muscle samples from a consistent anatomical site were allocated for muscle texture analysis; samples designated for compositional analysis, Cu^2+^ quantification, and mRNA expression assessment were flash-frozen in liquid nitrogen and maintained at −80 °C; and muscle tissue for histological sections was preserved in 4% paraformaldehyde.

### 2.4. Equations for Growth Performance

Growth performance was evaluated based on the following indices, which were calculated over the 56-day feeding trial [35,48]:

The following equations were used:

WGR (%) = [(W_t_ − W_0_)/W_0_] × 100

SGR (%/day) = [(lnW_t_ − lnW_0_)/56] × 100

FCR = Feed consumed/(W_t_ − W_0_)

Protein efficiency ratio (PER) = Weight Gain (g)/Protein Intake (g)

Lipid efficiency ratio (LER) = Weight Gain (g)/Lipid Intake (g)

Protein productive value (PPV) = Body Protein Gain (g)/Protein Intake (g)

Lipid productive value (LPV) = Body Lipid Gain (g)/Lipid Intake (g)

Survival rate (SR) (%) = The Number of Surviving Crayfish per Concrete Pond/25 × 100

Hepatosomatic index (HSI) (%) = Hepatopancreas wet weight/W_t_ × 100

Meat ratio (MR) (%) = Net meat weight/W_t_ × 100

Condition factor (CF) (g/cm^3^) = W_t_/final body length^3^ × 100

W_t_, final body weight; W_0_, initial body weight.

### 2.5. Proximate Composition and Cu^2+^ Concentration Analysis

The proximate composition (moisture, ash, crude protein, crude fat, and crude fiber) of feeds and crayfish muscle samples was determined using the standard methods of AOAC International [49]. Measurements were performed using eight biological replicates per experimental group. Total carbohydrates were calculated by difference. The gross energy levels of feeds were tested by the combustion method in an IKA C6000 oxygen bomb calorimeter (IKA WORKSGUANGZHOU, Guangzhou, China).

The quantification of Cu^2+^ concentrations in feeds, hepatopancreas, hemolymph, and muscle tissues was conducted utilizing an inductively coupled plasma mass spectrometer (ICP-MS: Agilent 7800, Agilent Technologies Inc., Santa Clara, CA, USA), adhering to the China National Food Safety Standard GB 5009.268-2016 [50]. Measurements were performed using four biological replicates per experimental group. Briefly, a precise aliquot (1 mL or 1 g) of each sample was mixed with 10 mL of nitric acid in a digestion vessel. After sealing, the vessel was positioned within the digestion apparatus for a pre-digestion phase at 120 °C for 30 min. A subsequent microwave-assisted digestion was executed with a temperature gradient: ramping to 130 °C and holding for 3 min; then to 150 °C for a 10 min hold; and finally to 180 °C for a 30 min hold. Once cooled to 60 °C, the digestate was transferred to a hot plate to drive off the acids until near dryness. The resulting residue was reconstituted with a 1% nitric acid solution to a final volume of 25 mL and thoroughly homogenized prior to analysis. The operational parameters for the ICP-MS system were configured as follows: pump speed at 20 r/min, nebulizer gas flow rate of 1.00 L/min, auxiliary gas flow rate of 1.00 L/min, a sample flush duration of 40 s, and an RF power setting of 1550 W.

### 2.6. Determination of Hemolymph Biochemical Indices and Antioxidant Indicators

Determinations of hemolymph biochemical profiles and antioxidant status were performed on eight biological replicates per group. The determination of hemolymph biochemical indices, including alkaline phosphatase (ALP), alanine aminotransferase (ALT) and aspartate aminotransferase (AST) activities, together with total protein (TP), albumin (ALB), globulin (GLB), triglyceride (TG), total cholesterol (TC) and glucose (GLU) concentrations, was quantified utilizing a Mindray BS-400 automatic biochemical analyzer (Shenzhen Mindray Bio-Medical Electronics Co., Ltd., Shenzhen, China) [35]. Additionally, HC concentration was determined using the ultraviolet absorption method. Briefly, hemolymph samples were diluted 10-fold, and the absorbance was measured at 335 nm using a Thermo Fisher Scientific Multiskan GO UV-Vis spectrophotometer (Model 1510, Waltham, MA, USA). The concentration was calculated according to the method described by Chen [51]. Total superoxide dismutase (T-SOD, A001-2-1), Cu/Zn-SOD (A001-2-1) and lysozyme (LZM, A050-1-1) activities, as well as PO (H247-1-2) and MDA (A003-1-1) concentrations, were assessed with corresponding commercial assay kits [35,52]. All experimental protocols were strictly adhered to as specified by the manufacturer. To guarantee measurements fell within the detection limits of the kits, a preliminary experiment was performed to establish the ideal sample volume. Every diagnostic kit applied in this study was supplied by Jiancheng Bioengineering Institute (Nanjing, China).

### 2.7. Texture Analysis and Determination of Muscle Hydroxyproline (HYP) Content

A dorsal muscle section (8 mm × 8 mm × 6 mm) was excised from each crayfish and analysed in the fresh (wet) state for texture parameters using a TA-XT plusC texture analyzer (Stable Micro Systems, Godalming, Surrey GU7 1YL, UK). The texture characteristics of muscle tissue, including hardness, springiness, cohesiveness, gumminess, chewiness, and resilience, were analyzed using a cylindrical stainless steel probe (P/50; diameter: 50 mm), with eight biological replicates per experimental group. The probe settings were: pre-test speed: 1 mm/s, test speed: 1 mm/s, post-test speed: 5 mm/s, trigger force: 20 g, and target deformation: 50% of the sample height. Shear force texture parameters were measured using an A-MORS probe, with settings of: pre-test speed: 1.5 mm/s, post-test speed: 10 mm/s, and trigger force: 20 g. Texture Profile Analysis (TPA) parameters were then calculated using the accompanying software (version 1.0) [53].

The hydroxyproline (HYP) content in muscle tissues was quantified using a commercial assay kit (A030-2-1, Jiancheng Bioengineering Institute, Nanjing, China), which is based on a standardized alkaline hydrolysis and colorimetric method [54]. Measurements were performed using eight biological replicates per experimental group. Briefly, muscle samples underwent alkaline hydrolysis to liberate HYP from collagen polypeptides. The liberated HYP was then oxidized with chloramine-T to form a pyrrole derivative, which subsequently reacted with 4-(dimethylamino)benzaldehyde (DMAB) at 60 °C to produce a chromophore with peak absorbance at 550 nm. The absorbance was measured using a spectrophotometer, and the HYP concentration was determined by interpolation from a standard curve prepared with known concentrations of HYP. It should be noted that the conversion factor of 8 can be used as a reasonable estimate for collagen content in muscle tissues of various animal species, including aquatic animals, due to the conserved high abundance of HYP in fibrillar collagens. This conversion factor (Collagen ≈ HYP × 8) is widely adopted in biochemical studies of meat products and is even recognized by official food composition regulations [55,56].

### 2.8. Muscle Histological Analysis

For histological analysis, muscle tissue sections were stained, with three biological replicates per experimental group. A small specimen of muscle tissue was harvested from each biological replicate and fixed in 4% paraformaldehyde buffer for 24 h. The fixed samples were then dehydrated through a graded ethanol series (30% for 2 h, 50% for 1 h, 75% for 80 min, 85% for 40 min, 95% for 30 min, 100% for 30 min, followed by a second incubation in 100% ethanol for 30 min). Specimens were then cleared by immersion in a 1:1 ethanol–xylene solution for 30 min, followed by two changes of pure xylene (30 min each). Following clearance, tissues were embedded in paraffin and sectioned at 5 μm thickness using a microtome (Leica RM2255, Nussloch, Germany). Sections were deparaffinized, rehydrated, and stained with hematoxylin and eosin (H.E.) according to the protocol described by Chen [57]. For each sample, five random fields of view were captured under a light microscope (50×, Olympus BX53; Olympus, Tokyo, Japan) equipped with a digital camera (Olympus DP73; Olympus, Tokyo, Japan). The cross-sectional area of individual muscle fibers was quantified using ImageJ software (version 1.53e; National Institutes of Health, Bethesda, MD, USA) by fitting each fiber to a circular model. Fiber diameter (μm) was subsequently derived from the calculated area (S = πr^2^, d = 2r). The size distribution of myofibers was categorized into three groups (<30 µm, 30–50 µm, >50 µm) following the established protocol of Valente [58].

### 2.9. Real-Time PCR Analysis

Real-time PCR analysis was performed using eight biological replicates per experimental group. Muscle tissue samples, preserved at −80 °C, were retrieved from each experimental group. Total RNA was extracted from the tissue using RNAiso Plus (Takara, Dalian, China). The concentration and quality of the RNA were determined using a NanoDrop 2000 spectrophotometer (Thermo Fisher Scientific, USA) with software version 1.6.0. All RNA samples were then diluted with diethyl pyrocarbonate (DEPC)-treated water to a uniform concentration of 500 ng/μL. cDNA was synthesized using the HiScript III RT SuperMix for qPCR (+gDNA wiper) (Vazyme, Nanjing, China) in a total volume of 20 μL. Briefly, 2 μL of total RNA was mixed with 4 μL of 4× gDNA wiper mix and 10 μL of RNase-free H_2_O. This mixture was incubated at 42 °C for 2 min to remove genomic DNA. Subsequently, 4 μL of 5× HiScript III qRT SuperMix was added, and the reverse transcription reaction was carried out at 37 °C for 15 min, followed by enzyme inactivation at 85 °C for 5 s.qPCR was performed using TB Green Premix Ex Taq II (Takara, Dalian, China) on a CFX 96 Real-time PCR Detection System (Bio-Rad, ver. 5.3.022.1030, Hercules, CA, USA). The 20 μL reaction mixture consisted of 10 μL of TB Green Premix, 0.8 μL of each primer (10 μM), 6.4 μL of ddH_2_O, and 2 μL of cDNA template. The thermal cycling protocol was as follows: initial denaturation at 95 °C for 30 s; followed by 30 cycles of denaturation at 95 °C for 5 s, annealing/extension at 60 °C for 30 s, and a final extension at 72 °C for 30 s. The temperature range of the dissolution curve was 50–95 °C [59].

All primers were designed based on the coding sequences (CDS) from the *P. clarkii* transcriptome database of our laboratory, utilizing online tools from NCBI (Bethesda, MD, USA), and were commercially synthesized by Shanghai Gene-ray Biotech Co., Ltd. (Shanghai, China, sequences provided in Table 2). The expression stability of all candidate reference genes was assessed with the geNorm algorithm. Primer efficiency and single-peak melting curves were verified before selecting an appropriate reference gene based on the average M value. The amplification efficiencies for some target genes exceeded 110%. It is recognized that amplification efficiencies between 90% and 110% are generally considered optimal. However, after rigorous validation including standard curve analysis (R^2^ > 0.99), distinct single-peak melting curves, and consistent repeatability across replicates, these primer sets were deemed acceptable for reliable relative quantification in the present study. Similar efficiency values have been reported and accepted in prior studies focusing on crustacean gene expression, where primer design is occasionally constrained by species-specific genomic sequences. The genes encoding *AK* and *EIF* were selected as endogenous reference controls. Their expression stability was validated using the geNorm algorithm, and normalization was performed based on the geometric mean of their expression levels. Relative gene expression levels were calculated using the 2^−ΔΔCt^ method [60]. The M values of the reference genes and the amplification efficiencies of the primers can be found in Figures S1 and S2.

### 2.10. Statistical Analysis

Data are presented as mean ± standard error of the mean (SEM). The normality of all data groups was first assessed using the Shapiro–Wilk test, and the homogeneity of variances was confirmed with Levene’s test. Subsequently, a one-way analysis of variance (ANOVA) was performed on all data using SPSS software (version 20.0; IBM Corp., Armonk, NY, USA). When the ANOVA indicated a significant effect, post hoc comparisons were conducted using Duncan’s new multiple range test. Based on the relationships identified from WGR and FCR data, broken-line analysis (piecewise linear regression) was carried out using OriginPro 2026 SR1 (Version 10.3, Build 10.300197; OriginLab Corporation, Northampton, MA, USA) to estimate the optimal dietary Cu^2+^ level. Differences were considered statistically significant at *p* < 0.05.

## 3. Results

### 3.1. Growth Performance

**Figure 1:**
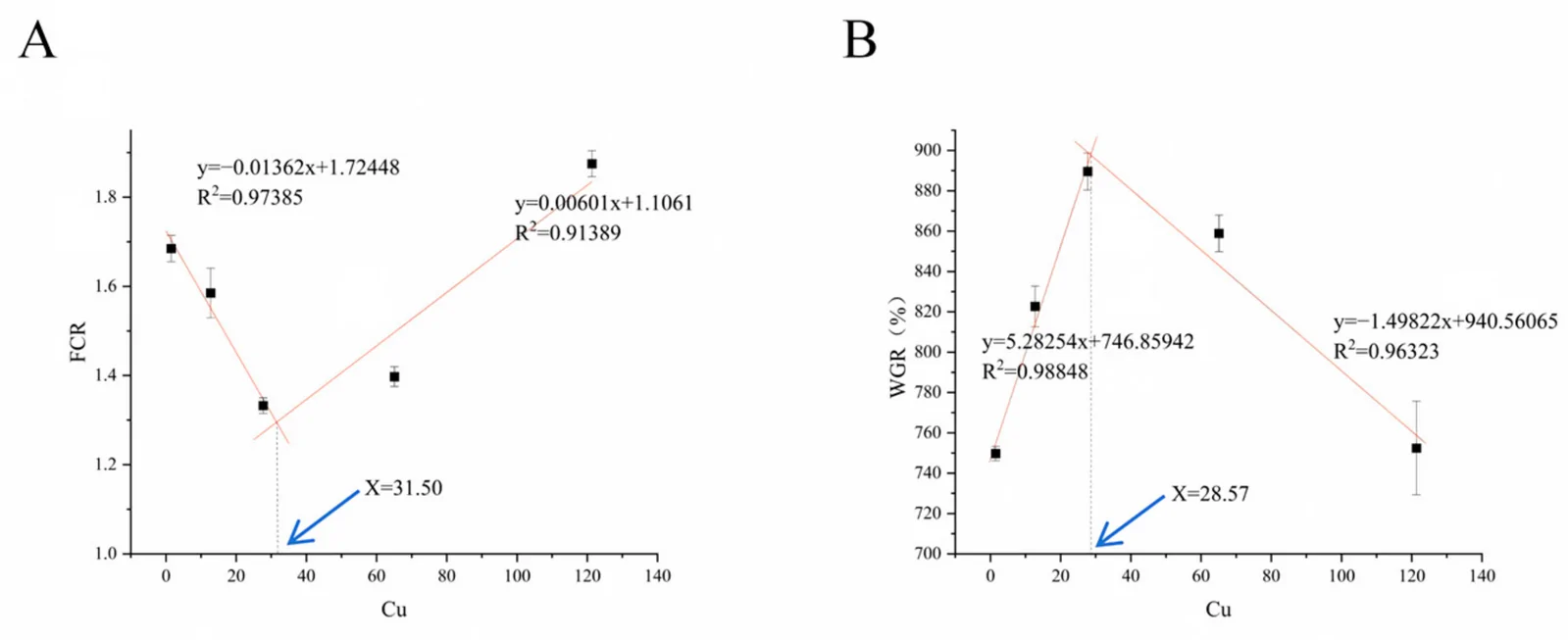
Relationship between dietary Cu^2+^ concentrations and WGR (**A**) and FCR (**B**) of juvenile *P. clarkii*. Note: WGR, weight gain rate; FCR, feed conversion ratio. The data were expressed as mean ± standard error (mean ± SEM).

Table 3 details the effects of dietary Cu^2+^ on the growth of *P. clarkii*. We observed that after 8 weeks, WGR and SGR were significantly higher (*p* < 0.05) in crayfish receiving 12.72, 27.71, or 65.09 mg/kg Cu^2+^ compared to those in 1.48 mg/kg Cu^2+^. Among them, crayfish fed the diet containing 27.71 mg/kg Cu^2+^ exhibited the highest final body weight (FBW), SGR, and WGR, along with the lowest FCR, these values differed significantly (*p* < 0.05) from those recorded at both the lowest (1.48 mg/kg) and the highest (121.34 mg/kg) Cu^2+^ concentrations. Meanwhile, the PER, LER, PPV, and LPV in the 27.71 mg/kg group were significantly higher than those in the 1.48 and 121.34 mg/kg groups (*p* < 0.05). Nevertheless, Cu^2+^ did not affect the HSI, MR and CF of juvenile crayfish (*p* > 0.05). No overt differences in external appearance were observed among treatments; crayfish in all groups displayed normal body coloration, intact appendages and a well-calcified carapace, and no gross signs of abnormality or of copper toxicity were seen at any sampling point. As shown in Figure 1, broken-line regression analysis based on weight gain rate (WGR) and feed conversion ratio (FCR) was used to estimate the optimal dietary Cu^2+^ requirement. For FCR, the two linear segments were y_1_ = −0.01362x + 1.72448 (R^2^ = 0.97) and y_2_ = 0.00601x + 1.10610 (R^2^ = 0.91), with a breakpoint at 31.50 mg/kg. For WGR, the corresponding segments were y_1_ = 5.28254x + 746.85942 (R^2^ = 0.99) and y_2_ = −1.49822x + 940.56065 (R^2^ = 0.96), yielding a breakpoint at 28.57 mg/kg. Because FCR decreased and then increased whereas WGR exhibited the opposite trend, both breakpoints represent the respective optima, indicating an optimal dietary copper requirement of 28.57–31.50 mg/kg.

### 3.2. Proximate Composition of Muscle

According to the results presented in Table 4, the moisture and crude ash content in the muscle of crayfish were not significantly altered by dietary Cu^2+^ inclusion (*p* > 0.05). The crude protein content was significantly influenced by dietary Cu^2+^ concentrations, with the 27.71 mg/kg group exhibiting a significantly higher value compared to all other groups (*p* < 0.05). Conversely, a statistically significant inverse pattern was observed for the muscle’s crude lipid content (*p* < 0.05).

### 3.3. Cu^2+^ Concentration in Tissues

**Figure 2:**
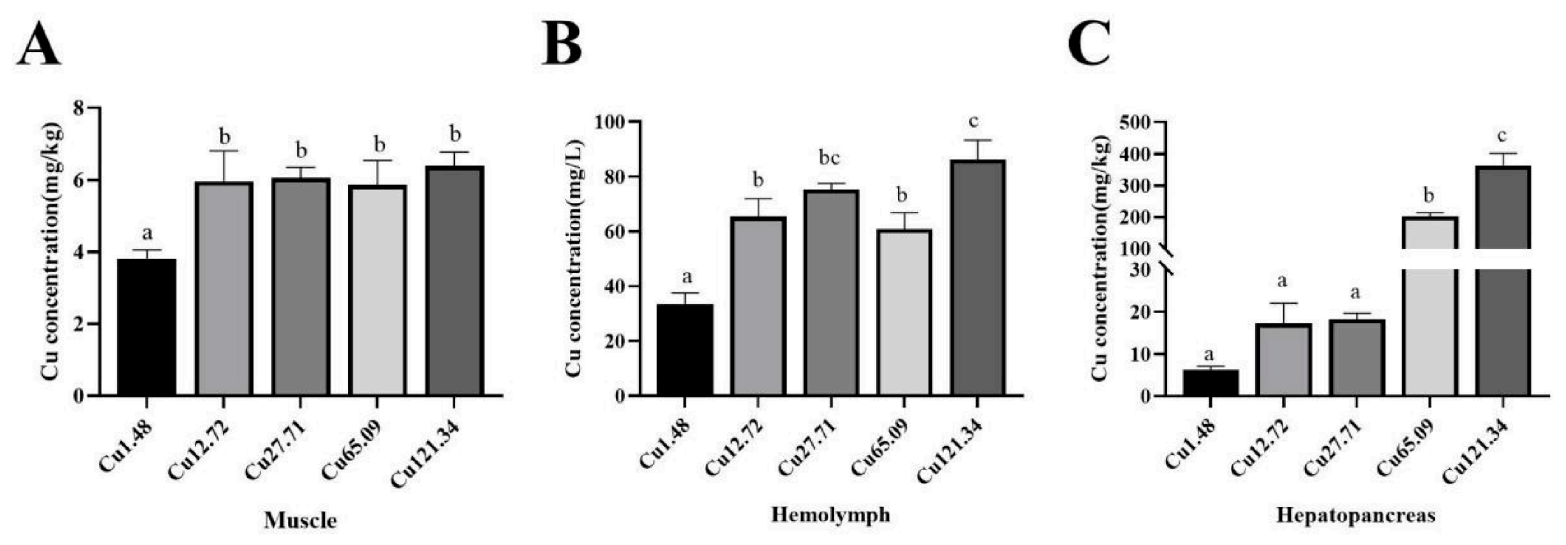
Cu^2+^ concentration in tissues of juvenile *P. clarkii* fed diets containing different Cu^2+^ concentrations. Note: (**A**) Cu^2+^ concentration in muscle; (**B**) Cu^2+^ concentration in hemolymph; (**C**) Cu^2+^ concentration in hepatopancreas. Different superscript letters indicate significant differences (*p* < 0.05). The data were expressed as mean ± standard error (mean ± SEM, *n* = 4).

Cu^2+^ concentrations in crayfish tissues are presented in Figure 2. Dietary Cu^2+^ concentrations significantly influenced Cu^2+^ concentrations in the muscle, hemolymph and hepatopancreas of crayfish (*p* < 0.05). No significant differences in muscle Cu^2+^ concentration were observed among the 12.72, 27.71, and 65.09 mg/kg diet groups (*p* > 0.05); however, all these groups exhibited significantly higher concentrations than the 1.48 mg/kg diet group (*p* < 0.05). The hemolymph Cu^2+^ concentration in the 121.34 mg/kg diet group was significantly higher than that in all other groups (*p* < 0.05). Furthermore, Cu^2+^ concentrations in the hepatopancreas were significantly elevated in the 65.09 and 121.34 mg/kg diet groups compared to the 1.48, 12.72, and 27.71 mg/kg diet groups (*p* < 0.05).

### 3.4. Hemolymph Biochemical Parameters

**Figure 3:**
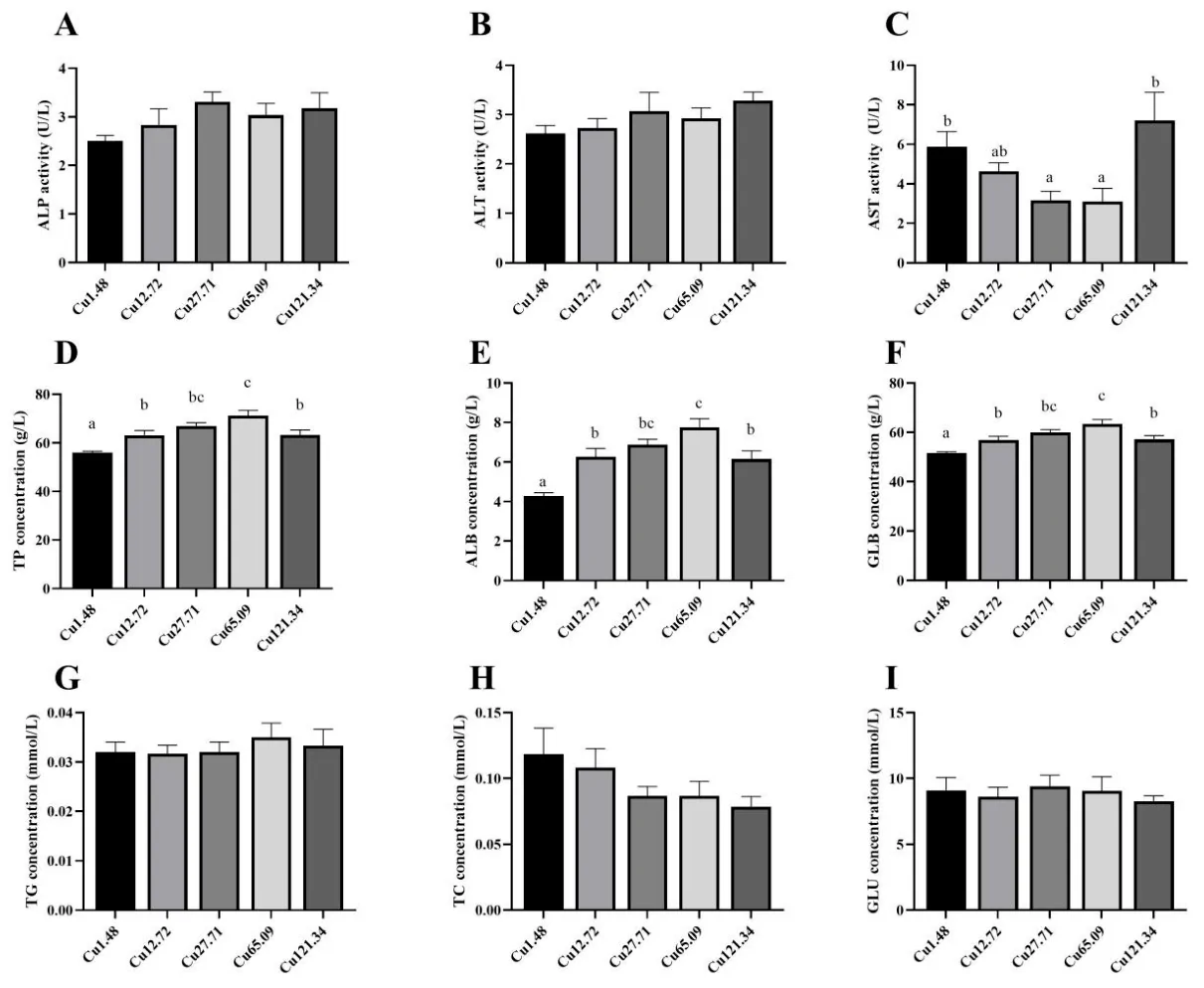
Effects of dietary Cu^2+^ concentration on hematological parameters of juvenile *P. clarkii*. Note: (**A**) ALP, alkaline phosphatase; (**B**) ALT, alanine aminotransferase; (**C**) AST, aspartate aminotransferase; (**D**) TP, total protein; (**E**) ALB, albumin; (**F**) GLB, globulin; (**G**) TG, triglyceride; (**H**) TC, cholesterol; (**I**) GLU, glucose. Different superscript letters indicate significant differences (*p* < 0.05). The data were expressed as mean ± standard error (mean ± SEM, *n* = 8).

Hemolymph biochemical parameters are depicted in Figure 3. Compared to the 1.48 and 121.34 mg/kg diet groups, AST activities were significantly lower in crayfish fed diets containing 27.71 or 65.09 mg/kg Cu^2+^ (*p* < 0.05). The peak concentrations of TP, ALB, and GLB were observed in the 65 mg/kg group, showing significant differences compared to the 1.48, 12.72, and 121.34 mg/kg groups (*p* < 0.05). No significant differences were observed among groups for the activities of ALP and ALT, or the concentrations of TG, TC, and GLU (*p* > 0.05).

### 3.5. Antioxidant Parameters

**Figure 4:**
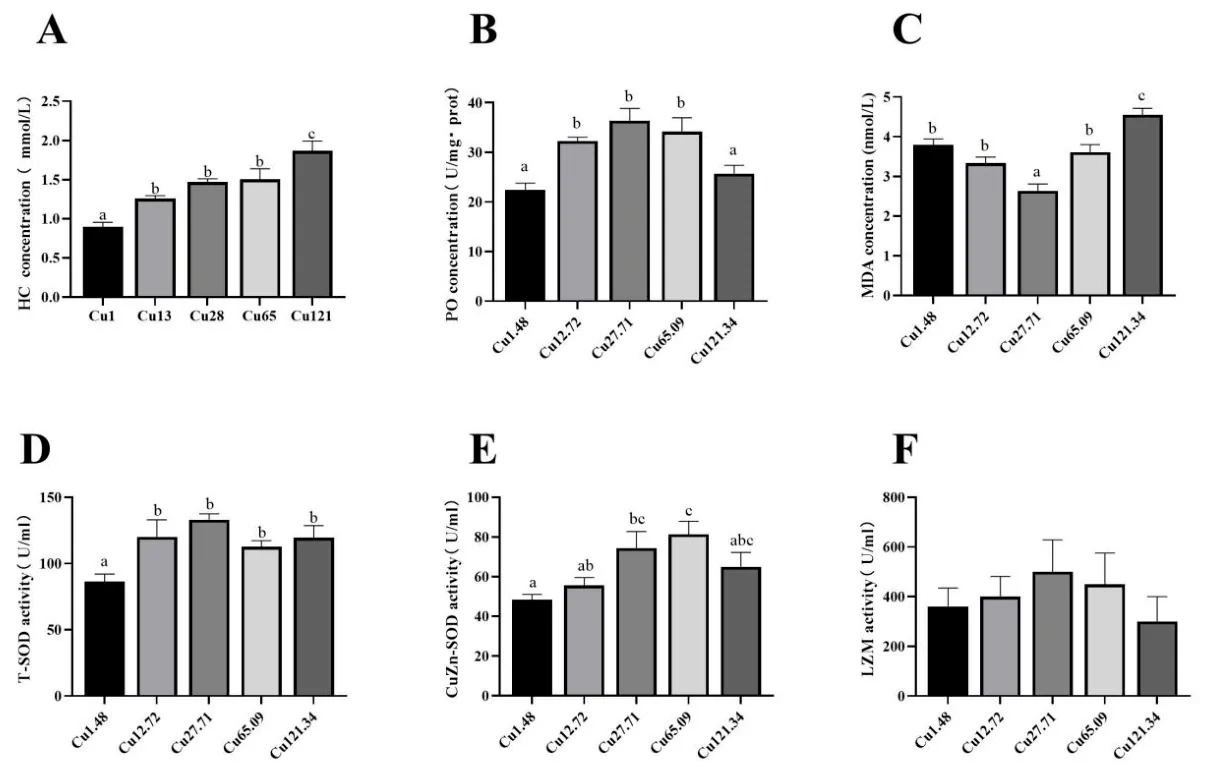
Effects of dietary Cu^2+^ concentration on antioxidant capacity in hemolymph of juvenile *P. clarkii*. Note: (**A**) HC, hemocyanin; (**B**) PO, phenoloxidase; (**C**) MDA, malondialdehyde; (**D**) T-SOD, total-superoxide dismutase; (**E**) Cu/Zn-SOD, Cu/Zn-superoxide dismutase; (**F**) LZM, lysozyme. Different superscript letters indicate significant differences (*p* < 0.05). The data were expressed as mean ± standard error (mean ± SEM, *n* = 8).

The hemolymphatic antioxidant profile is presented in Figure 4. The concentration of dietary Cu^2+^ exerted a significant influence on the activities of T-SOD, Cu/Zn-SOD, and the concentrations of PO, HC and MDA (*p* < 0.05). A progressive elevation in HC concentrations was observed with higher dietary Cu^2+^, with all treatment groups showing notably higher values than the Cu^2+^ concentration of 1.48 mg/kg group (*p* < 0.05). The maximum concentration for PO and activity for T-SOD were recorded at the 27.71 mg/kg dose, which were significantly greater than those in the lowest Cu^2+^ group (*p* < 0.05). A significant difference in MDA concentration was found among the dietary Cu^2+^ concentrations (*p* < 0.05). The concentration was minimal in the 27.71 mg/kg group and was statistically distinct from that of every other group (*p* < 0.05). No statistically significant variations in LZM activity were detected across the different dietary Cu^2+^ concentrations (*p* > 0.05).

### 3.6. Muscle Texture Profile Analysis (TPA) and Hydroxyproline Content

**Figure 5:**
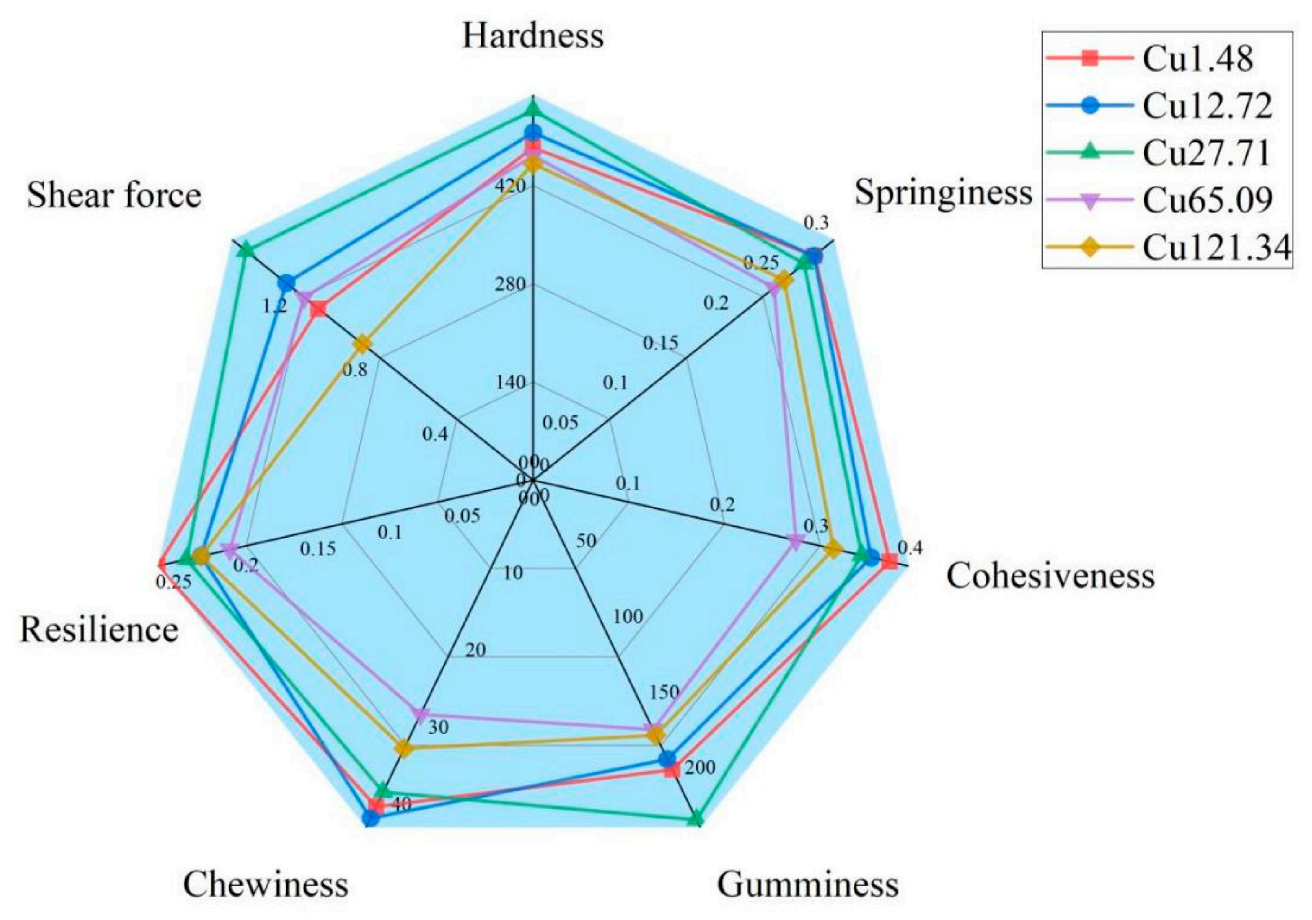
Effects of dietary Cu^2+^ concentration on muscle texture profile analysis of juvenile *P. clarkii*.

**Figure 6:**
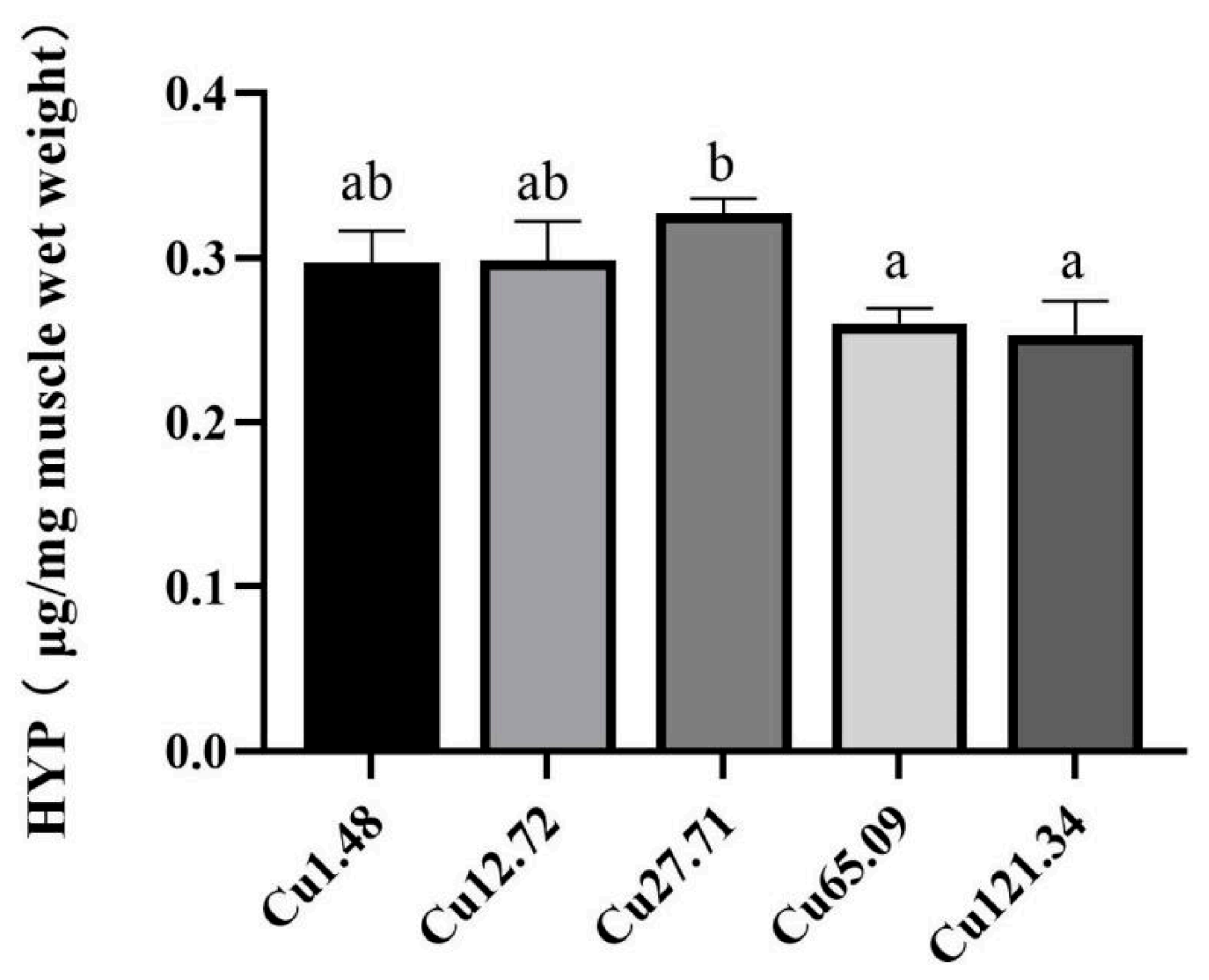
Effects of dietary Cu^2+^ concentration on HYP in muscle of juvenile *P. clarkii*. Note: HYP, hydroxyproline. Different superscript letters indicate significant differences (*p* < 0.05). The data were expressed as mean ± standard error (mean ± SEM, *n* = 8).

The muscle textural properties of *P. clarkii* are shown in Table 5 and Figure 5 and Figure 6. The hardness, gumminess, and shear force of the crayfish fed the 27.71 mg/kg diet group were the highest. There was an initial rise and a subsequent decline in HYP concentrations as the dietary Cu^2+^ concentration increased, with the maximum value being observed at 27.71 mg/kg. Concentrations at this peak concentration (27.71 mg/kg) exhibited significant differences (*p* < 0.05) compared with those in both the 1.48 and 121.34 mg/kg dietary groups.

### 3.7. Histomorphometric Analysis of Muscle Tissue

**Figure 7:**
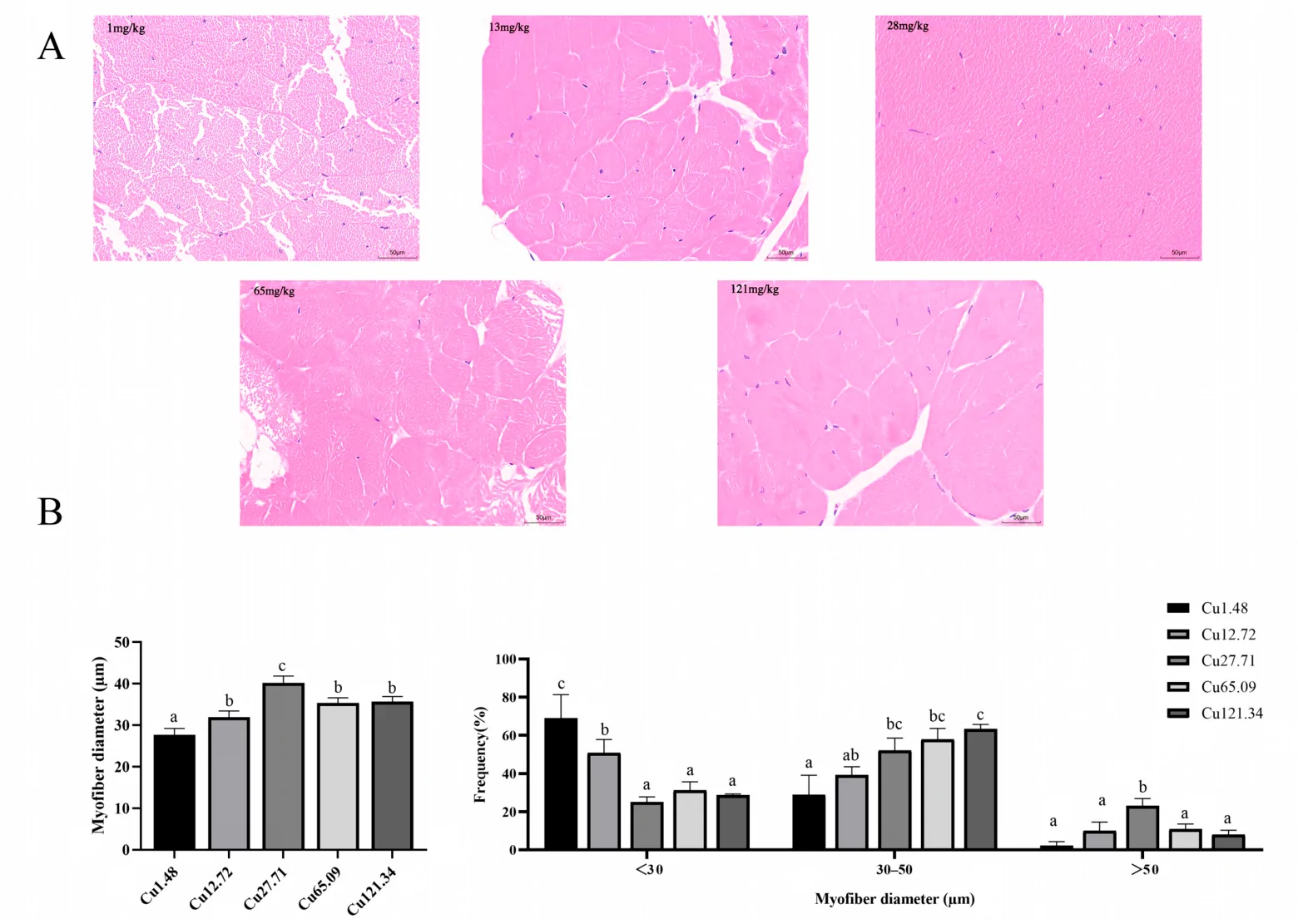
Effects of dietary Cu^2+^ concentration on the histological morphology of muscle in juvenile *P. clarkii*. Note: (**A**) Representative histological images of muscle sections stained with Hematoxylin and Eosin (H&E). Scale: 50 µm; magnification: 40×. (**B**) Quantitative analysis of myofiber diameter. Different superscript letters indicate significant differences (*p* < 0.05). The data were expressed as mean ± standard error (mean ± SEM, *n* = 3).

As demonstrated in Figure 7, dietary Cu^2+^ concentrations influence the myofiber characteristics of juvenile *P. clarkii*. The average muscle fiber diameter was markedly elevated in the dietary groups receiving 12.72, 27.71, 65.09, and 121.34 mg/kg of Cu^2+^ compared to the 1.48 mg/kg group (*p* < 0.05). Furthermore, a significantly higher prevalence of muscle fibers exceeding 50 μm in diameter was observed specifically in the cohort fed with 27.71 mg/kg Cu^2+^ relative to all other treatments (*p* < 0.05).

### 3.8. Gene Expression

#### 3.8.1. Cu^2+^ Homeostasis

**Figure 8:**
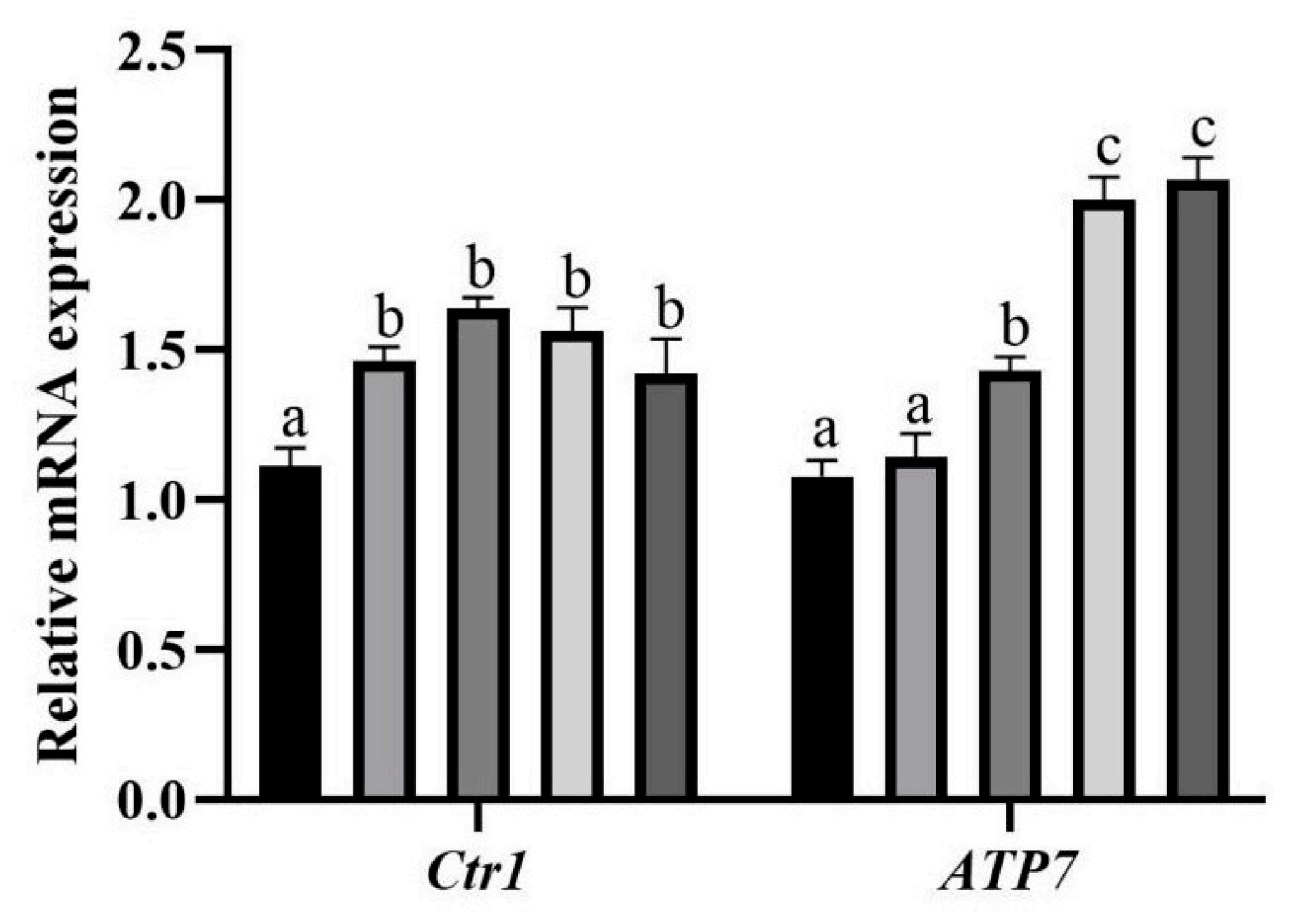
Effects of dietary Cu^2+^ concentration on relative mRNA expression of genes involved in Cu^2+^ homeostasis in muscle of juvenile *P. clarkii*. Note: *Ctr1*, Copper Transport protein 1; *ATP7*, copper-transporting ATPase. Different superscript letters indicate significant differences (*p* < 0.05). The data were expressed as mean ± standard error (mean ± SEM, *n* = 8).

The mRNA abundance of genes associated with Cu^2+^ homeostasis is depicted in Figure 8. Dietary Cu concentrations markedly modulated the muscle transcript levels of pivotal genes responsible for Cu^2+^ influx (*Ctr1*) and efflux (*ATP7*). Specifically, *Ctr1* expression was significantly elevated in the 12.72, 27.71, and 65.09 mg/kg Cu^2+^ groups relative to the 1.48 mg/kg group (*p* < 0.05). *ATP7* expression was notably upregulated across other treatment groups when compared to the 1.48 and 12.72 mg/kg groups (*p* < 0.05).

#### 3.8.2. Collagen Synthesis

**Figure 9:**
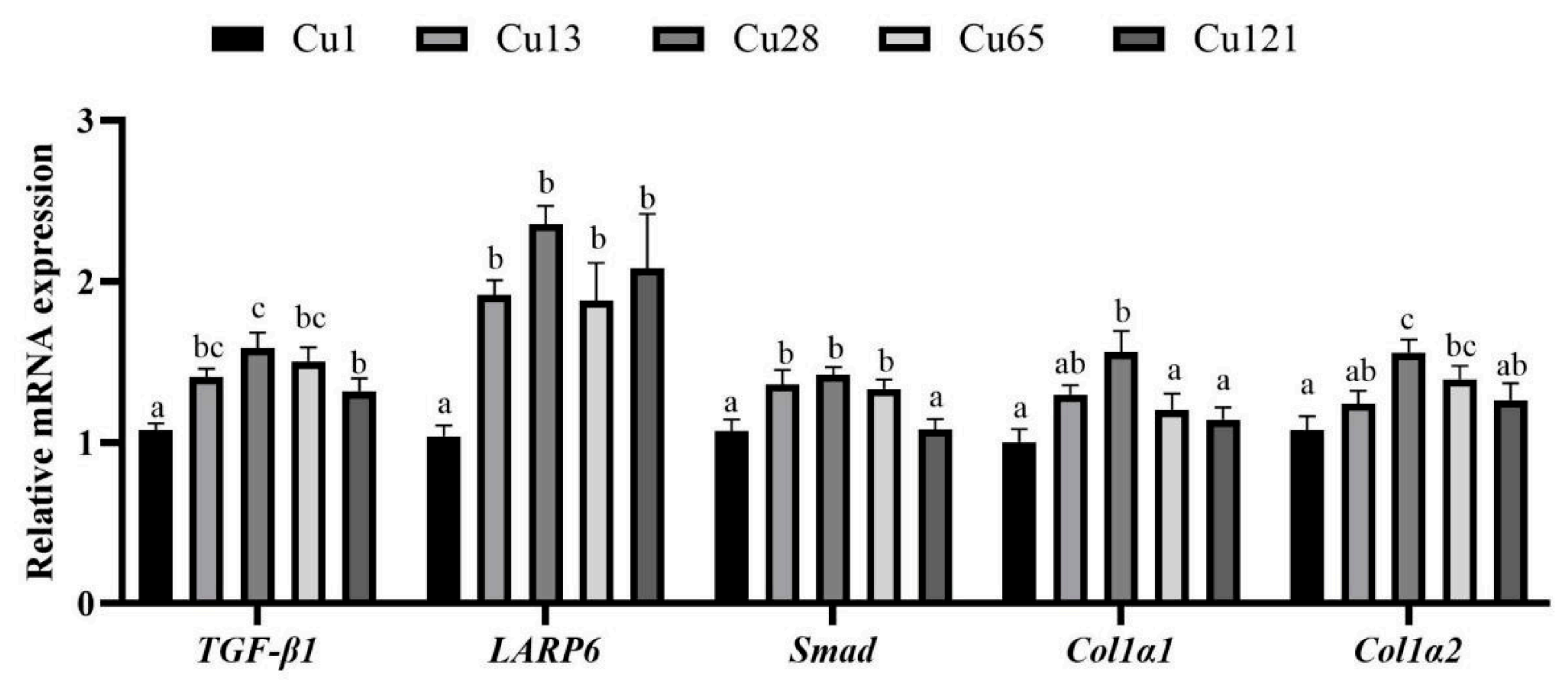
Effects of dietary Cu^2+^ concentration on relative mRNA expression of genes involved in collagen in muscle of juvenile *P. clarkii*. Note: *TGF-β1*, transforming growth factor-β1; *LARP6*, la ribonucleoprotein 6; *Col1α1*, type I collagen α1; *Col1α2*, type I collagen α2. Different superscript letters indicate significant differences (*p* < 0.05). The data were expressed as mean ± standard error (mean ± SEM, *n* = 8).

As depicted in Figure 9, the transcript levels of collagen-associated genes (*TGF-β1*, *LARP6*, *Smad*, *col1α1* and *col1α2*) displayed a biphasic response to increasing dietary Cu^2+^. Their expression first rose and then declined, reaching a maximum in the group fed the 27.71 mg/kg diet. At this peak concentration, the expression was significantly higher than that in the 1.48 mg/kg diet group (*p* < 0.05).

#### 3.8.3. Muscle Development

**Figure 10:**
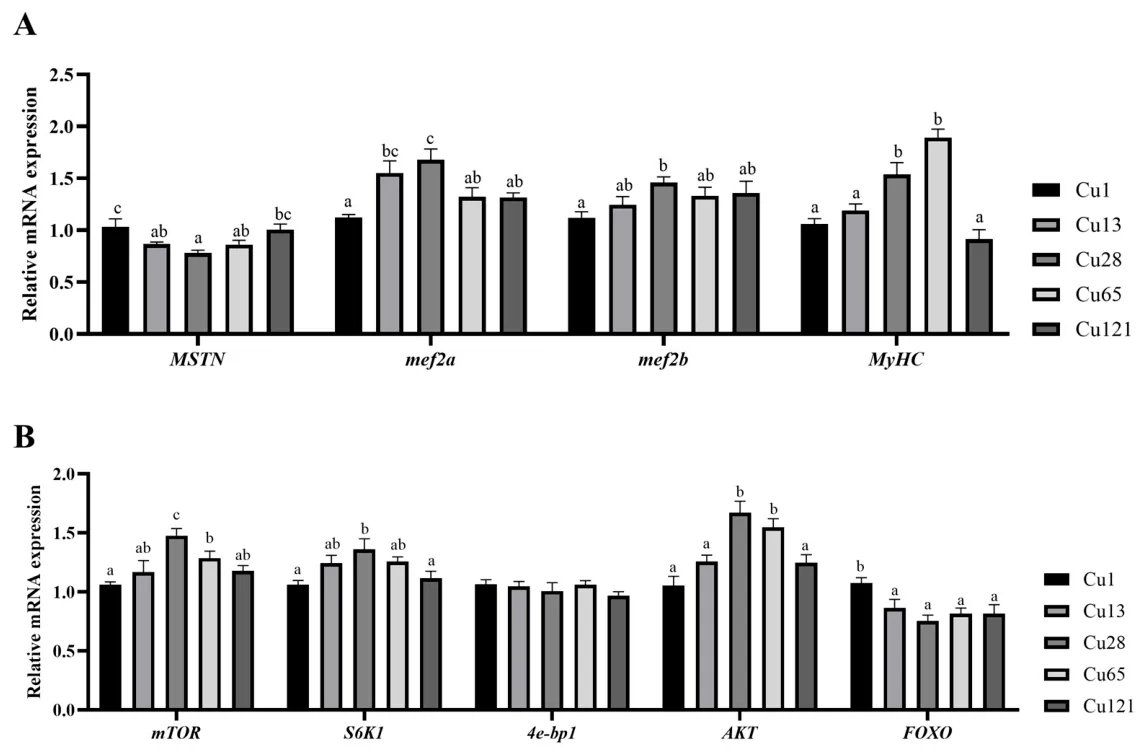
Effects of dietary Cu^2+^ concentration on relative mRNA expression of genes involved in muscle development of juvenile *P. clarkii*. Note: (**A**) Relative expression of genes related to myosins and muscle regulatory factors; (**B**) Relative expression of genes related to protein synthesis, signaling molecules and transcription factors. *MSTN*, myoSTatin; *mef2a*, myocyte enhancer factor 2A; *mef2b*, myocyte enhancer factor 2B; *MyHC*, myosin heavy chain; *mTOR*, mammalian target of rapamycin; *S6K1*, ribosomal protein subunit 6 kinase 1; *4e-bp1*, eIF4E-binding protein 1; *AKT*, protein kinase B; *FOXO*, forkhead box O. Different superscript letters indicate significant differences (*p* < 0.05). The data were expressed as mean ± standard error (mean ± SEM, *n* = 8).

As illustrated in Figure 10, the mRNA abundance of genes associated with muscle development exhibited distinct patterns in response to dietary Cu^2+^. The expression of *mef2a*, *mef2b*, *MyHC*, *mTOR*, *S6K1* and *AKT* demonstrated a biphasic response, rising initially and then declining as Cu^2+^ concentrations increased, with the peak expression observed in the 27.71 mg/kg group. The highest expression level of *MyHC* was observed at a dose of 65.09 mg/kg. This peak value was markedly elevated relative to that in the 1.48 mg/kg group (*p* < 0.05). Conversely, the transcript levels of *MSTN* and *FOXO* showed an opposite trend, decreasing first and then increasing, and their lowest expression was recorded in the 27.71 mg/kg diet group, which was significantly suppressed compared to the 1.48 mg/kg group (*p* < 0.05). There was no significant difference in *4e-bp1* expression among the groups (*p* > 0.05).

## 4. Discussion

### 4.1. Overall Effect of Dietary Cu^2+^ Concentrations on the Growth Performance of P. clarkii

In aquaculture species, weight gain rate (WGR), specific growth rate (SGR), and feed conversion ratio (FCR) serve as reliable proxies for evaluating dietary nutrient balance. Cu^2+^, as one of the trace elements essential for the growth of aquatic organisms, plays a critical role in their development [19]. In this study, at a dietary Cu^2+^ supplementation of 27.71 mg/kg, the WGR, SGR, and FCR of crayfish were optimal. This observation aligns with findings from analogous studies in other crustaceans. Sun [64] reported that during an 8-week feeding trial, dietary Cu^2+^ at 40.3 mg/kg enabled Chinese mitten crab (*Eriocheir sinensis*) to achieve optimal growth performance. Similarly, dietary Cu^2+^ at 26–27 mg/kg enabled oriental river prawn (*Macrobrachium nipponense*) to achieve maximum WGR over an eight-week culture period when fed a semi-purified diet [65]. Collectively, these results indicate that growth performance is compromised when dietary Cu^2+^ supply is either suboptimal or excessive, whereas an adequate supply is beneficial. When dietary Cu^2+^ supply falls below the requirement, the function of key cuproenzymes such as cytochrome c oxidase and lysyl oxidase leads to a cascade of physiological disorders including anemia, energy metabolism dysfunction, skeletal abnormalities, and oxidative stress, which ultimately results in reduced growth performance in animals [66,67,68]. Conversely, when dietary Cu^2+^ is excessive, Cu^2+^ accumulates in tissues such as the hepatopancreas, which can cause tissue damage and ultimately impair growth performance [69]. De Boeck proposed that under Cu^2+^-exposed conditions, animals allocate more energy toward excretory metabolism of excess Cu^2+^, repair of tissue damage and development of defense mechanisms, leaving less energy available for growth, thereby resulting in growth reduction [70]. Based on a broken-line regression model applied to the WGR and FCR data, the optimal dietary Cu^2+^ inclusion concentration for juvenile *P. clarkii* was estimated to be between 28.57 and 31.50 mg/kg.

### 4.2. Cu^2+^ Accumulation in Tissues and Homeostatic Regulation

Cu^2+^ in crustaceans is derived from both ambient water and the diet, with the dietary contribution being the dominant origin [27]. Tissue Cu^2+^ accumulation increases commensurately with dietary Cu^2+^ levels, a relationship corroborated by multiple studies [11,71,72]. Cu^2+^ concentrations in the muscle, hemolymph, and hepatopancreas of *P. clarkii* exhibited a positive correlation with dietary Cu^2+^ supplementation. Studies in yellow catfish (*Pelteobagrus fulvidraco*) [73,74], red sea bream (*Pagrus major*) [75], Pacific white shrimp (*Litopenaeus vannamei*) [76], and Oriental river prawn (*Macrobrachium nipponense*) [64] have shown that the hepatopancreas is the main site for Cu^2+^ storage in aquatic animals. It can counteract Cu^2+^ toxicity by upregulating the expression of genes associated with immune response, metabolism, and detoxification [77]. When aquatic animals ingest excess Cu^2+^, excess Cu^2+^ can accumulate in the hepatopancreas through the blood to alleviate the toxicity caused by high Cu^2+^; when Cu^2+^ intake is insufficient, the Cu^2+^ stored in the hepatopancreas can be utilized by the body [78]. In this experiment, after 8 weeks of culture, the hepatopancreas Cu^2+^ contents in crayfish from the 65.09 and 121.34 mg/kg Cu-supplemented groups were significantly higher than those in the other groups.

Dietary Cu^2+^ absorption occurs predominantly in the intestine [79]. A set of evolutionarily conserved proteins controls Cu^2+^ import, export, and intracellular utilization, thereby maintaining Cu^2+^ ion concentrations within a certain range [8]. Key regulators include high-affinity copper uptake protein 1 (*Ctr1*), ATPase copper transporting polypeptide (*ATP7*), copper chaperone for superoxide dismutase (*CCS*), antioxidant 1 copper chaperone (*ATOX1*), metal regulatory transcription factor 1/2 (*MTF-1/2*), metallothionein (*MT*), and glutathione (GSH) [42]. Among them, the membrane transporter *Ctr1* (also known as SLC31A1) is primarily responsible for facilitating Cu^2+^ uptake into cells, whereas *ATP7* (including *ATP7A* and *ATP7B*) promotes Cu^2+^ efflux [80]. To investigate the precise regulatory mechanisms, we examined the expression of Cu^2+^ transporter genes *Ctr1* and *ATP7* in muscle. *Ctr1* is essential for transcellular Cu^2+^ translocation [22] and mediates extracellular Cu^2+^ import. Studies have reported that intracellular Cu^2+^ levels directly regulate *Ctr1*mRNA expression in small cell lung cancer (SCLC) cells; lower Cu^2+^ concentrations promote its expression, whereas higher concentrations exert an inhibitory effect [81]. The results demonstrated that the expression level of the *Ctr1* gene in *P. clarkii* muscle exhibited a biphasic response, initially rising and subsequently declining in response to elevating dietary Cu^2+^ concentrations. This may be because increased intracellular Cu^2+^ concentrations reduce the transcription level of *Ctr1* mRNA, leading to a decrease in *Ctr1* protein synthesis, thereby negatively regulating the intracellular Cu^2+^ concentration through feedback to avoid the organism absorbing excessive Cu^2+^, preventing Cu^2+^ poisoning from excessive Cu entering the cell. *ATP7* is mainly responsible for Cu^2+^ ion efflux from cells [23], and it has two main types, *ATP7A* and *ATP7B*. When Cu^2+^ concentration in cells gradually increases, the ATPase protective mechanism is activated; *ATP7A* and *ATP7B* carry Cu^2+^ and undergo conformational changes in the protein structural region under kinase phosphorylation conditions [82,83]. The mechanism of the relationship between Cu^2+^ concentration and *ATP7* distribution and transport is consistent. Excess Cu^2+^ can induce *ATP7* redistribution, ensuring rapid and effective Cu^2+^ efflux [84]. In this study, with increasing dietary Cu^2+^ concentrations, the expression of the *ATP7* gene in muscle significantly increased. The above suggests that muscle regulates Cu^2+^ homeostasis through Cu^2+^ influx and efflux, which also explains why muscular Cu^2+^ accumulation in crayfish exhibited no significant variation across a spectrum of dietary Cu^2+^ supplementation concentrations ranging from 12.72 to 121.34 mg/kg.

### 4.3. Effects of Cu^2+^ on Hemolymph Physiology, Biochemistry, and Antioxidant Function

Blood physiological and biochemical indicators in aquatic animals provide an integrated reflection of their physiological status, metabolism, nutritional status, and disease conditions [85]. Hemolymph AST and ALT activities are widely used as indicators of hepatopancreatic cell damage in crustaceans [86]. In this study, AST in the hemolymph of crayfish in the 27.71 mg/kg and 65.09 mg/kg Cu^2+^ groups was significantly lower than in the 1.48 mg/kg and 121.34 mg/kg addition groups. This pattern suggests that both Cu^2+^ deficiency and Cu^2+^ excess induced hepatopancreatic injury. It should be noted, however, that AST is not hepatopancreas-specific and no histopathological examination of the hepatopancreas was conducted in this study; the above interpretation is therefore tentative. The hepatopancreas is the main source of hemolymph proteins, and hemolymph TP is commonly used as an index of protein metabolic and nutritional status in crustaceans [87]. In this study, TP and ALB increased and then declined with increasing dietary Cu^2+^, peaking at intermediate Cu^2+^ levels, which may reflect a better protein metabolic status at an adequate Cu^2+^ supply. These changes should not, however, be interpreted as evidence of enhanced protein synthesis or immunity. Hemolymph protein concentration in crustaceans varies with nutritional status, molt stage and hemolymph volume, and the concurrent Cu-dependent increase in HC may itself have contributed to the elevated TP.

The assessment of oxidative status must first rely on the endpoint marker of damage. MDA is the end product of oxidative damage; its increased concentration directly reflects the severity of oxidative stress in the organism and is the most commonly used indicator for evaluating lipid peroxidation in aquatic animals [88]. In the present study, the MDA content in hemolymph showed an initial decrease followed by an increase as dietary Cu^2+^ levels increased, reaching the lowest value in the 27.71 mg/kg group, which was significantly lower than that in the other groups. This phenomenon can be fundamentally explained by the dual role of Cu^2+^ in redox biology: at optimal concentrations, Cu^2+^ acts as an essential cofactor for antioxidant enzymes, whereas at excessive levels, it catalyzes the generation of highly reactive hydroxyl radicals via the Fenton reaction, leading to severe lipid peroxidation and membrane damage [8,89]. To further explore this phenomenon, we investigated Cu-containing enzymes and proteins such as HC, PO, and SOD. HC is a multifunctional Cu-containing protein in mollusks and arthropods [9]. Its main roles include oxygen transport and storage, as well as involvement in osmoregulation, molting cycle, exoskeleton formation, and melanin synthesis [90]. Although HC is traditionally recognized for its role in oxygen transport, it has been increasingly acknowledged for its involvement in antioxidant defense and immune responses in crustaceans [91,92]. Therefore, the alteration in HC concentration observed in this study may also reflect the modulatory effect of dietary Cu^2+^ on the systemic antioxidant capacity of the crayfish. As an essential component of HC, Cu supplementation within an appropriate range can increase HC content, thereby promoting the growth and development of crustaceans. A study on *Eriocheir sinensis* reported that the best growth performance and a significant increase in hemolymph HC content were observed at dietary Cu^2+^ concentrations of 20.78–40.34 mg/kg [64]. However, when dietary Cu^2+^ exceeds the metabolic requirement, the surplus Cu^2+^ accumulates in the hepatopancreas and hemolymph, disrupts Cu^2+^ homeostasis and induces oxidative stress, thereby imposing a toxic challenge on the crayfish. As a component of the antioxidant and immune defense system in crustaceans, HC is correspondingly upregulated in response to such Cu-induced toxicity. In mud crab (*Scylla paramamosain*), a high-Cu diet (162 mg/kg) led to abnormal coloration of the carapace and hemolymph, along with upregulated HC expression [93]. Consistent with these findings, the present study showed that HC content increased with increasing dietary Cu^2+^. The HC levels in the 12.72, 27.71, and 65.09 mg/kg groups were significantly higher than that in the 1 mg/kg group, but significantly lower than that in the 121.34 mg/kg group. PO, a Cu-containing protein, is found ubiquitously across diverse organisms including bacteria, fungi, plants, and animals [94]. Functionally, it is pivotal to melanogenesis and constitutes a fundamental element of the crustacean innate immune system [95]. Studies have shown that insufficient Cu^2+^ intake restricts PO synthesis, while excessive Cu^2+^ induces intensified oxidative stress, overwhelming PO and leading to reduced concentration [96]. Mechanistically, PO requires two Cu ions (Cu^2+^) at its active site to bind oxygen and perform its enzymatic functions [44]. Thus, adequate dietary Cu^2+^ ensures the synthesis of PO. In this study, when the dietary Cu^2+^ addition concentration was 27.71 mg/kg, the PO concentration in the hemolymph of crayfish was the highest. As a crucial antioxidant enzyme, SOD serves a vital function in cellular defense against oxidative damage caused by free radicals [97]. In animal systems, the Cu/Zn-SOD form represents a primary antioxidant enzyme, constituting most of the T-SOD activity. Its activity level can serve as an excellent indicator for evaluating the Cu^2+^ nutritional status of animals [98]. The mechanism underlying this is explicit: Cu/Zn-SOD strictly requires Cu^2+^ as a cofactor to catalyze the dismutation of superoxide radicals into hydrogen peroxide and oxygen [93]. Therefore, the dietary supplementation of 27.71 mg/kg Cu^2+^ optimally satisfied the cofactor demand of Cu/Zn-SOD, maximizing its enzymatic activity to eliminate harmful superoxides and consequently suppressing MDA. Conversely, both Cu^2+^ deficiency and excess compromised this delicate balance. In this study, hemolymph concentrations of both T-SOD and Cu/Zn-SOD in crayfish exhibited initial elevation followed by decline with increasing dietary Cu^2+^ supplementation. The pattern of T-SOD and Cu/Zn-SOD activity alterations may be analogous to that observed for PO, where a dietary Cu^2+^ supply below the requirement restricts synthesis, whereas excessive Cu^2+^ intensifies oxidative stress, consequently constraining enzymatic activity. In summary, this indicates that appropriate dietary Cu^2+^ can effectively improve oxidative stress in juvenile *P. clarkii*, which is consistent with studies in aquatic animals such as *Litopenaeus vannamei* [99] and *Pagrus major* [75].

### 4.4. Improvement in Muscle Structure, Chemical Composition and Function by Dietary Cu^2+^ and the Underlying Molecular Mechanisms

Muscle structure, chemical composition and function are crucial biological traits for aquatic animals, closely related to nutrient deposition, contractile performance, and structural integrity. This study evaluated the effect of Cu^2+^ on the muscle structure, chemical composition and function of *P. clarkii* from multiple dimensions, including nutritional composition, protein metabolism, and textural properties.

#### 4.4.1. Myofiber Development

As the fundamental unit of muscle, the structure and growth of myofibers directly influence muscle structure [100]. A 9-week feeding trial by Ma et al. demonstrated that dietary Cu^2+^ supplementation at 4.06 mg/kg significantly enhanced the body weight and myofiber diameter of juvenile grass carp, promoting muscle growth [32], which is consistent with our findings. In this study, dietary Cu supplementation at 27.71 mg/kg increased the mean myofiber diameter in juvenile crayfish. This effect was characterized by a reduced frequency of myofibers with a diameter < 30 μm and an elevated frequency of those > 50 μm, indicating that appropriate dietary Cu^2+^ can effectively promote myofiber hypertrophy in juvenile crayfish. However, further increasing the dietary Cu^2+^ concentration led to a reduction in the measured indicators related to myofiber growth and development in juvenile crayfish, although these values remained higher than those at 1.48 mg/kg Cu^2+^.

This phenomenon may be attributed to alterations in the expression of genes such as *MSTN*, which are related to myosin and muscle regulatory factors. *MSTN* negatively regulates myofiber growth by inhibiting muscle hyperplasia, partly through suppression of the *mTOR* signaling pathway [38]. The myocyte enhancer factor-2 (*mef2a*, *mef2b*) family plays a crucial role as regulators of myofiber formation [40]. *MyHC* encodes a molecular motor that influences contraction velocity and muscle performance and is considered a marker for myofiber type [39]. In this study, the relative expression level of *MSTN* was observed to decrease initially and then increase with rising dietary Cu^2+^ concentrations. Conversely, the expression levels of *mef2a*, *mef2b*, and *MyHC* exhibited an opposite trend, initially increasing before decreasing. The coordinated changes in the transcript levels of *MSTN*, *mef2a*, *mef2b*, and *MyHC* suggest that moderate dietary Cu^2+^ may be associated with the promotion of myofiber growth and development in crayfish.

#### 4.4.2. Muscle Nutrient Deposition

The deposition of nutrients primarily refers to the content of protein and fat within muscle tissue. In the present study, the crude protein content was highest in the group supplemented with 27.71 mg/kg dietary Cu^2+^. This finding aligns with research in *Ctenopharyngodon idella* [32] and *Pagrus major* [75], indicating that an appropriate concentration of dietary Cu^2+^ can enhance protein deposition in muscle. This phenomenon may result from the combined effect that Cu^2+^ at an appropriate level activates the TGF-β1/Smads signaling pathway and upregulates myogenic regulatory factors such as myoblast determination protein 1 (*MyoD*), myogenic factor 5 (*Myf5*), and myogenin (*MyoG*), thereby promoting collagen deposition in muscle and the proliferation and differentiation of muscle fibers, ultimately leading to increased muscle protein deposition [32]. Studies have shown that muscle lipid content in aquatic organisms decreases with increasing dietary Cu^2+^ [11,101]. Consistent with this, the crude fat content in muscle was lowest in the 27.71 mg/kg dietary Cu^2+^ group in our study. This effect may be attributed to the ability of dietary Cu^2+^ to stimulate lipolysis and β-oxidation [76].

Protein deposition is a fundamental process for muscle growth and development, achieved through both myofiber proliferation and hypertrophy [102]. Ultimately, the balance struck between synthetic and degradatory protein metabolic pathways governs the degree of protein deposition and the resultant muscle mass. mTOR, belonging to the phosphatidylinositol 3-kinase-related kinase (PIKK) family [103], is a key signaling regulator of muscle protein synthesis. It is activated by phosphorylation at Ser2448 via the phosphatidylinositol 3-kinase (PI3K)/AKT signaling pathway [104]. Factors such as AKT activation lead to mTOR activation [105], which subsequently phosphorylates downstream molecules S6K1 and 4e-bp1. This results in increased transcription of mRNA encoding ribosomal proteins and elongation factors, promoting ribosome recruitment and translation initiation [106], thereby enhancing protein synthesis. The group receiving a dietary Cu^2+^ level of 27.71 mg/kg exhibited pronounced upregulation in the mRNA expression of *mTOR*, *S6K1* and *AKT*, showing a significant elevation compared to all other treatment groups. This coordinated upregulation of key components of the AKT/mTOR signaling pathway is associated with the enhanced muscle protein deposition observed at this Cu^2+^ level. Therefore, the improved protein accretion may be, at least in part, mediated through the AKT/mTOR signaling cascade. Future investigations quantifying phosphorylated protein levels or employing targeted pathway modulators are warranted to substantiate this mechanistic link.

There are two primary pathways responsible for regulating protein degradation: the ubiquitin–proteasome system and the autophagy–lysosome pathway [107]. FOXO participates in the regulation of autophagy and influences protein degradation processes [41]. In the present study, the observed downregulation of *FOXO* mRNA expression in the 27.71 mg/kg dietary Cu^2+^ group was concurrent with increased muscle protein content. Given the established role of *FOXO* transcription factors in regulating protein degradation pathways, this correlation suggests an association between dietary Cu^2+^, *FOXO* transcript abundance, and protein accretion; whether Cu^2+^ modulates FOXO activity at the protein level, and hence proteolysis, remains to be determined. Further studies are needed to confirm whether Cu^2+^ directly modulates FOXO activity at the protein level and its downstream effects on proteolysis.

#### 4.4.3. Muscle Hydroxyproline Content as an Estimate of Collagen Deposition

Beyond influencing myofiber development and promoting protein synthesis, dietary Cu^2+^ may also contribute to collagen deposition (estimated via HYP) in muscle. Our results showed that appropriate dietary Cu^2+^ (27.71 mg/kg) significantly increased HYP content. This aligns with findings in *Ctenopharyngodon idella*, where dietary Cu^2+^ similarly enhanced HYP-based collagen estimates [32].

Type I collagen is the predominant collagenous component in muscle, composed of α1 and α2 peptide chains (*Col1α1* and *Col1α2*) [108]. Its expression increases with elevated expression of *TGF-β1* and *Smad* [36]. In our study, the relative mRNA expression levels of *TGF-β1*, *Smad*, *Col1α1*, and *Col1α2* were coordinately highest in the 27.71 mg/kg dietary Cu^2+^ group, which aligned with the peak in muscle HYP content. At the transcript level, this co-expression pattern is consistent with the involvement of the TGF-β1/Smads signaling axis in collagen regulation, as reported in mice by Je-Won Ko [109]. The concomitant upregulation of these genes suggests a regulatory association between dietary Cu^2+^ intake and the transcriptional network governing collagen synthesis in crayfish muscle. *LARP6*, which belongs to the superfamily of RNA-binding proteins, mediates the translation initiation of *Col1α1* and *Col1α2* mRNA post-transcription [37,110]. It is noteworthy that the 27.71 mg/kg dietary group exhibited the peak relative expression of the *LARP6* gene, showing a marked contrast to the 1.48 mg/kg group. The observed upregulation of *LARP6* at the transcriptional level suggests that Cu^2+^ might influence the post-transcriptional regulation of collagen synthesis. However, further studies at the protein and functional levels are required to confirm its role in translation initiation.

To unequivocally establish causality, our future studies will include functional validation experiments, such as gene knockdown (e.g., *LARP6* knockdown) or targeted inhibition of the TGF-β1/Smads pathway, to test the proposed mechanistic link between Cu^2+^ and collagen regulation.

#### 4.4.4. Muscle Texture

Muscle texture, which reflects the physicochemical properties of the tissue, is another key indicator for evaluating muscle quality. Hardness and shear force are two central textural parameters, and both are positively correlated with myofiber diameter and density [111] as well as with the content, distribution, and cross-linking degree of collagen [19,112]. In the present study, dietary Cu^2+^ at 27.71 mg/kg increased instrumental hardness, gumminess, and shear force, an effect that may be attributable to the concurrent increases in average myofiber diameter and muscle collagen content. Previous work has shown that an enlarged average myofiber diameter, together with increased collagen deposition, markedly enhances the hardness of grass carp muscle [113].

In shrimp, greater hardness and shear force are generally regarded as indicators of improved texture, although this relationship is not absolute. On the one hand, a moderate increase in hardness and shear force may be beneficial for crayfish: it enhances perceived firmness and elasticity, reduces textural disintegration during handling, cooking, and transport, and may help compensate for the soft, underdeveloped musculature commonly observed in small *P. clarkii*. On the other hand, once firmness exceeds an optimal threshold, it is usually accompanied by reduced tenderness and juiciness and, consequently, lower overall acceptability; in red claw crayfish (*Cherax quadricarinatus*), for example, a higher shear force coincided with lower sensory scores for tenderness, juiciness, and overall acceptability [114]. Because no sensory evaluation or consumer test was conducted in this study, we interpret our findings as a copper-induced modification of muscle texture and composition rather than as unambiguous evidence of improved muscle quality.

## 5. Conclusions

Dietary Cu at 27.71 mg/kg improved growth performance, antioxidant capacity and muscle quality in juvenile *P. clarkii*, accompanied by coordinated changes in the transcript levels of genes governing myofiber growth (*MSTN*, *MyHC*, *mef2a*, *mef2b*) and collagen synthesis (*TGF-β1*, *Smad*, *Col1α1*, *Col1α2*, *LARP6*). These changes should be read as transcriptional correlates of the phenotypic response rather than as evidence of pathway activation.

Based on broken-line regression of WGR and FCR, the optimal dietary Cu requirement of juvenile *P. clarkii* is estimated at 28.57–31.50 mg/kg. We therefore recommend that dietary copper be supplemented to a concentration within this range to support good growth, antioxidant capacity and muscle quality in juvenile *P. clarkii*. Concentrations above this range conferred no additional benefit and should be avoided.

## Figures and Tables

**Table 1:** Formulation of the experimental diet.

Ingredients (%)	Group				
	Cu1.48	Cu12.72	Cu27.71	Cu65.09	Cu121.34
Fish meal ^1^	16.5	16.5	16.5	16.5	16.5
Casein ^1^	25.0	25.0	25.0	25.0	25.0
Pregelatinized starch ^1^	33.0	33.0	33.0	33.0	33.0
Soybean oil	2.5	2.5	2.5	2.5	2.5
Monocalcium phosphate ^1^	2.0	2.0	2.0	2.0	2.0
Vitamin and mineral premix ^2^	2.0	2.0	2.0	2.0	2.0
Carboxymethyl cellulose ^1^	2.0	2.0	2.0	2.0	2.0
DMPT ^3^	2.0	2.0	2.0	2.0	2.0
Microcrystalline cellulose ^1^	13.0	13.0	13.0	13.0	13.0
Soybean lecithin ^1^	2.0	2.0	2.0	2.0	2.0
Total	100.0	100.0	100.0	100.0	100.0
CuSO_4_·5H_2_O ^4^	0	0.005	0.011	0.023	0.046
Measured Cu (mg/kg, as-fed)	1.48 ± 0.10	12.72 ± 0.18	27.71 ± 0.12	65.09 ± 0.12	121.34 ± 0.18
Proximate composition (%, DM)					
Moisture (%)	10.29 ± 0.15	10.13 ± 0.19	10.22 ± 0.14	10.31 ± 0.09	10.18 ± 0.11
Dry Matter (%)	89.71 ± 0.15	89.87 ± 0.19	89.78 ± 0.14	89.69 ± 0.09	89.82 ± 0.11
Crude Protein (%)	32.27 ± 0.17	32.32 ± 0.10	32.29 ± 0.15	32.31 ± 0.16	32.33 ± 0.17
Ether Extract (%)	6.11 ± 0.12	6.13 ± 0.16	6.09 ± 0.08	6.07 ± 0.12	6.10 ± 0.11
Crude Fiber (%)	14.75 ± 0.16	14.72 ± 0.15	14.77 ± 0.17	14.69 ± 0.16	14.80 ± 0.17
Ash (%)	6.72 ± 0.13	6.69 ± 0.16	6.75 ± 0.18	6.73 ± 0.12	6.71 ± 0.16
Gross energy (MJ/kg)	15.83 ± 0.09	15.79 ± 0.13	15.74 ± 0.09	15.79 ± 0.16	15.76 ± 0.13

Note: Analysed copper concentrations and proximate composition are presented as mean ± SD of three analytical replicates per diet (*n* = 3). ^1^ Obtained from Tongwei Co., Ltd. (Wuxi, China). ^2^ Vitamin and mineral premix was composed of a 1:1 mixture of vitamin premix and mineral premix. Provided the following per kilogram of vitamin premix: vitamin A, 4 g; vitamin D_3_, 0.02 g; vitamin E, 10 g; menadione (vitamin K_3_), 10 g; thiamine (vitamin B_1_), 10 g; riboflavin (vitamin B_2_), 10 g; pyridoxine (vitamin B_6_), 20 g; nicotinamide, 40 g; biotin, 0.2 g; calcium pantothenate, 20 g; folic acid, 0.5 g; cyanocobalamin (vitamin B_12_), 0.01 g; ascorbic acid (vitamin C), 20 g; inositol, 400 g. Provided the following per kilogram of mineral premix: potassium iodate, 0.6 g; sodium selenite pentahydrate, 0.08 g; monopotassium phosphate, 320 g; magnesium sulfate, 200 g; manganese sulfate monohydrate, 20 g; zinc sulfate heptahydrate, 60 g; ferrous sulfate heptahydrate, 50 g; sodium chloride, 100 g; cobalt chloride hexahydrate, 2 g; microcrystalline cellulose, 247.32 g. ^3^ DMPT: dimethyl-beta-propiothetin. ^4^ Cu sulfate pentahydrate (CuSO_4_·5H_2_O) was sourced from Shanghai McLean Biochemical Technology Co., Ltd. (Shanghai, China).

**Table 2:** Primer sequences for real-time PCR.

Gene	Primers (5′–3′)	Accession No.	R^2^	Amplification Efficiency (%)
*AK (F)*	TCCTCGACGTAATCCAGTCC	database ^1^	0.99	109.61
*AK (R)*	CGAAGTCCTTGTTGGGATGT			
*EIF (F)*	GGAATAAGGGGACGAAGACC	[61]	1.00	110.97
*EIF (R)*	GCAAACACACGCTGGGAT			
*Ctr1 (F)*	GCTAAAGCAGTTGTGGACGC	XM_069300064.1	1.00	109.66
*Ctr1 (R)*	GCTGTGGCCAACATGAACTG			
*ATP7 (F)*	GCCTTGAGCTCTGTGTCTGT	XM_069309993.1	0.99	110.85
*ATP7 (R)*	CGGTACTGGAATGTGGCCTT			
*TGF-β1 (F)*	TTGAGTTGGCAGAGCACAGT	XM_045727777.2	0.99	112.91
*TGF-β1 (R)*	TGGTTGAGCTCGCATGAACT			
*LARP6 (F)*	TCAACCGCTGCTCCTCCAAGAT	XM_045726349.2	1.00	112.80
*LARP6 (R)*	ACCGACTGTATGCTGGGCTTCT			
*Smad (F)*	ACCTGAGAGGCGAAGGAGAT	XM_045742441.2	1.00	105.95
*Smad (R)*	TGATGCAAGCACACGGGTAT			
*Col1α1 (F)*	CCGGACGAGTTGAAGGCAC	XM_069319039.1	1.00	109.60
*Col1α1 (R)*	CCGTAGGGTCCTCCCTTTG			
*Col1α2 (F)*	GCAGCGAGGTTGTTGCTGAA	XM_045729274.2	1.00	113.49
*Col1α2 (R)*	ACCCTTGCTGGTGGCTTCTT			
*MSTN (F)*	CGACTTGGGTTGGGACTTC	[62]	0.99	115.90
*MSTN (R)*	CCTTTGGTGCCACGATGA			
*mef2a (F)*	CATCTTCCAACCATCCTGGG	[62]	0.99	112.18
*mef2a (R)*	GTTTGCTCAACGGGGTATCA			
*mef2b (F)*	ACCAGCACCACCTTCACATT	[62]	0.99	98.88
*mef2b (R)*	GAAGATGGACCCAAATGTGAA			
*MyHC (F)*	AAGCCAACCGTACCCTCAA	[53]	1.00	109.93
*MyHC (R)*	AGTAGCACGTTCTCTGCATTCA			
*mTOR (F)*	GAAGGCATGCTGCGGTATTG	[61]	0.99	100.97
*mTOR (R)*	CGCAGGCTTTGGGTCTCTTA			
*S6k1 (F)*	ACAGCCGAGAATCGCAAGAA	[61]	1.00	107.60
*S6k1 (R)*	ATCACCATTATCGGGTCCGC			
*4e-bp1 (F)*	ACCTGCCAGTGATACCAGGA	[61]	1.00	106.82
*4e-bp1 (R)*	TGGCTCCTCTGAAATCGTTCC			
*AKT (F)*	CGTGGCAGTCTACGTAAGGT	[53]	0.99	109.46
*AKT (R)*	TACGAGTGGTTGCCCTTTGG			
*FOXO (F)*	ACGCGCTAACACCATGGAAG	[63]	0.99	103.27
*FOXO (R)*	GACTCTCACTCAGCGACGAA			

Note: ^1^ The sequence of AK was acquired from the *P. clarkii* RNAseq database. *AK*, arginine kinase; *EIF*, eukaryotic initiation factor; *Ctr1*, copper transport protein 1; *ATP7*, copper-transporting ATPase; *TGF-β1*, transforming growth factor-β1; *LARP6*, la ribonucleoprotein 6; *Col1α1*, type I collagen α1; *Col1α2*, type I collagen α2; *MSTN*, myoSTatin; *mef2a*, myocyte enhancer factor 2A; *mef2b*, myocyte enhancer factor 2B; *MyHC*, myosin heavy chain; *mTOR*, mammalian target of rapamycin; *S6K1*, ribosome protein subunit 6 kinase 1; *4e-bp1*, eIF4E-bind-ing protein 1; *AKT*, protein kinase B; *FOXO*, forkhead box O.

**Table 3:** Effects of dietary Cu^2+^ concentration on growth performance of juvenile *P. clarkii* over an 8-week feeding trial.

Items	Cu1.48	Cu12.72	Cu27.71	Cu65.09	Cu121.34	*p* Value
IBW (g)	1.83 ± 0.01	1.84 ± 0.01	1.84 ± 0.00	1.85 ± 0.01	1.84 ± 0.00	0.399
FBW (g)	15.51 ± 0.11 ^a^	16.95 ± 0.14 ^b^	18.21 ± 0.17 ^c^	17.69 ± 0.13 ^bc^	15.71 ± 0.44 ^a^	<0.001
WGR (%)	749.74 ± 3.58 ^a^	822.69 ± 10.11 ^b^	889.54 ± 9.18 ^c^	858.87 ± 9.06 ^bc^	752.45 ± 23.21 ^a^	<0.001
SGR (%/d)	3.82 ± 0.01 ^a^	3.97 ± 0.02 ^b^	4.10 ± 0.02 ^c^	4.04 ± 0.02 ^bc^	3.83 ± 0.05 ^a^	<0.001
FCR	1.69 ± 0.03 ^b^	1.59 ± 0.06 ^b^	1.33 ± 0.02 ^a^	1.40 ± 0.02 ^a^	1.88 ± 0.03 ^c^	<0.001
PER (%)	184.08 ± 3.22 ^b^	195.93 ± 6.86 ^b^	232.54 ± 3.07 ^c^	221.64 ± 3.52 ^c^	165.08 ± 2.55 ^a^	<0.001
LER (%)	972.21 ± 17.01 ^b^	1033.03 ± 36.15 ^b^	1232.94 ± 16.27 ^c^	1179.94 ± 18.72 ^c^	874.95 ± 13.54 ^a^	<0.001
PPV (%)	18.31 ± 0.49 ^ab^	19.74 ± 0.89 ^b^	25.94 ± 0.52 ^d^	24.08 ±0.56 ^c^	16.58 ± 0.43 ^a^	<0.001
LPV (%)	41.87 ± 1.09 ^b^	43.16 ± 1.94 ^b^	50.37 ± 1.85 ^c^	49.95 ± 0.44 ^c^	37.88 ± 0.38 ^a^	<0.001
SR (%)	86.00 ± 2.58	88.00 ± 1.63	87.00 ± 3.42	87.00 ± 2.52	89.00 ± 3.42	0.952
HSI (%)	7.14 ± 0.22	7.28 ± 0.26	6.75 ± 0.22	7.50 ± 0.29	7.75 ± 0.29	0.066
MR (%)	11.98 ± 0.53	11.61 ± 0.42	11.35 ± 0.50	11.04 ± 0.53	11.64 ± 0.52	0.745
CF	2.87 ± 0.06	3.00 ± 0.06	3.01 ± 0.07	2.99 ± 0.06	3.09 ± 0.06	0.213

Note: IBW, initial body weight; FBW, final body weight; WGR, weight gain rate; SGR, specific growth rate; FCR, feed conversion ratio; PER, protein efficiency ratio; LER, lipid efficiency ratio; PPV, protein productive value; LPV, lipid productive value; SR, survival rate; HSI, hepatosomatic index; MR, meat ratio; CF, condition factor. The approximate chemical composition of the entire body of the initial and final crayfish is shown in Supplementary Table S1. Different superscript letters indicate significant differences (*p* < 0.05). The data were expressed as mean ± standard error (mean ± SEM).

**Table 4:** Effects of diets containing graded concentrations of Cu^2+^ on the proximate composition of the muscle of juvenile *P. clarkii* over an 8-week feeding trial.

Items	Cu1.48	Cu12.72	Cu27.71	Cu65.09	Cu121.34	*p* Value
Moisture (%)	76.40 ± 0.35	76.20 ± 0.39	76.31 ± 0.15	76.48 ± 0.09	76.31 ± 0.58	0.986
Crude protein (%)	19.96 ± 0.09 ^a^	20.36 ± 0.09 ^b^	20.81 ± 0.03 ^c^	20.32 ± 0.06 ^b^	20.45 ± 0.02 ^b^	<0.001
Crude lipid (%)	1.08 ± 0.06 ^b^	1.08 ± 0.11 ^b^	0.70 ± 0.00 ^a^	0.93 ± 0.04 ^bc^	1.01 ± 0.09 ^b^	0.022
Ash (%)	1.96 ± 0.27	2.52 ± 0.19	1.95 ± 0.23	2.66 ± 0.25	2.62 ± 0.20	0.117

Note: Different superscript letters indicate significant differences (*p* < 0.05). The data were expressed as mean ± standard error (mean ± SEM, *n* = 8).

**Table 5:** Effects of dietary Cu^2+^ concentration on muscle texture profile analysis of juvenile *P. clarkii* over an 8-week feeding trial.

Items	Cu1.48	Cu12.72	Cu27.71	Cu65.09	Cu121.34	*p* Value
Hardness (N)	474.19 ± 12.35 ^ab^	495.90 ± 39.02 ^ab^	527.69 ± 12.47 ^b^	462.91 ± 12.25 ^a^	451.21 ± 12.75 ^a^	0.043
Springiness (mm)	0.28 ± 0.01	0.28 ± 0.01	0.27 ± 0.02	0.24 ± 0.02	0.25 ± 0.02	0.178
Cohesiveness	0.38 ± 0.01 ^b^	0.36 ± 0.01 ^b^	0.35 ± 0.03 ^ab^	0.28 ± 0.01 ^a^	0.32 ± 0.02 ^ab^	0.045
Gumminess (N)	191.62 ± 12.65 ^a^	184.75 ± 9.49 ^a^	224.92 ± 5.77 ^b^	165.11 ± 11.45 ^a^	168.86 ± 4.90 ^a^	<0.001
Chewiness (MJ)	42.30 ± 2.35 ^bc^	43.77 ± 3.25 ^c^	40.40 ± 2.14 ^bc^	30.33 ± 3.61 ^a^	34.77 ± 2.04 ^ab^	0.021
Resilience	0.26 ± 0.02	0.23 ± 0.01	0.24 ± 0.02	0.21 ± 0.02	0.23 ± 0.02	0.437
Shear force (N)	1.07 ± 0.06 ^b^	1.23 ± 0.07 ^bc^	1.43 ± 0.09 ^c^	1.14 ± 0.06 ^b^	0.85 ± 0.06 ^a^	<0.001

Note: Different superscript letters indicate significant differences (*p* < 0.05). The data were expressed as mean ± standard error (mean ± SEM, *n* = 8).

## Data Availability

The data supporting the results of this article will be made available by the corresponding authors on request.

## Supplementary Materials

The following supporting information can be downloaded at: https://www.mdpi.com/article/10.3390/ani16182949/s1, Figure S1: The reaction efficiency of different genes by qPCR analysis. The accuracy of real-time PCR assays was high (R^2^ > 0.99) for all diluents based on linear regression analysis; Figure S2: Ranking of candidate reference genes based on their expression stability measured by the geNorm algorithm. Table S1: Approximate chemical composition of the whole crayfish body at the initial and final stages.
