# Supplementary material for: Effects of Dietary Copper on Growth Performance, Antioxidant Capacity, Muscle Quality and the Expression of Copper-Transport and Muscle Development-Related Genes in Juvenile Red Swamp Crayfish (Procambarus clarkii)
Source: Animals (Basel). 2026 Sep 19;16(18):2949. doi: 10.3390/ani16182949 (PMC13603499; doi:10.3390/ani16182949)
Supplement: Supplementary file 1 [file animals-16-02949-s001.zip › animals-4538324-supplementary.pdf]

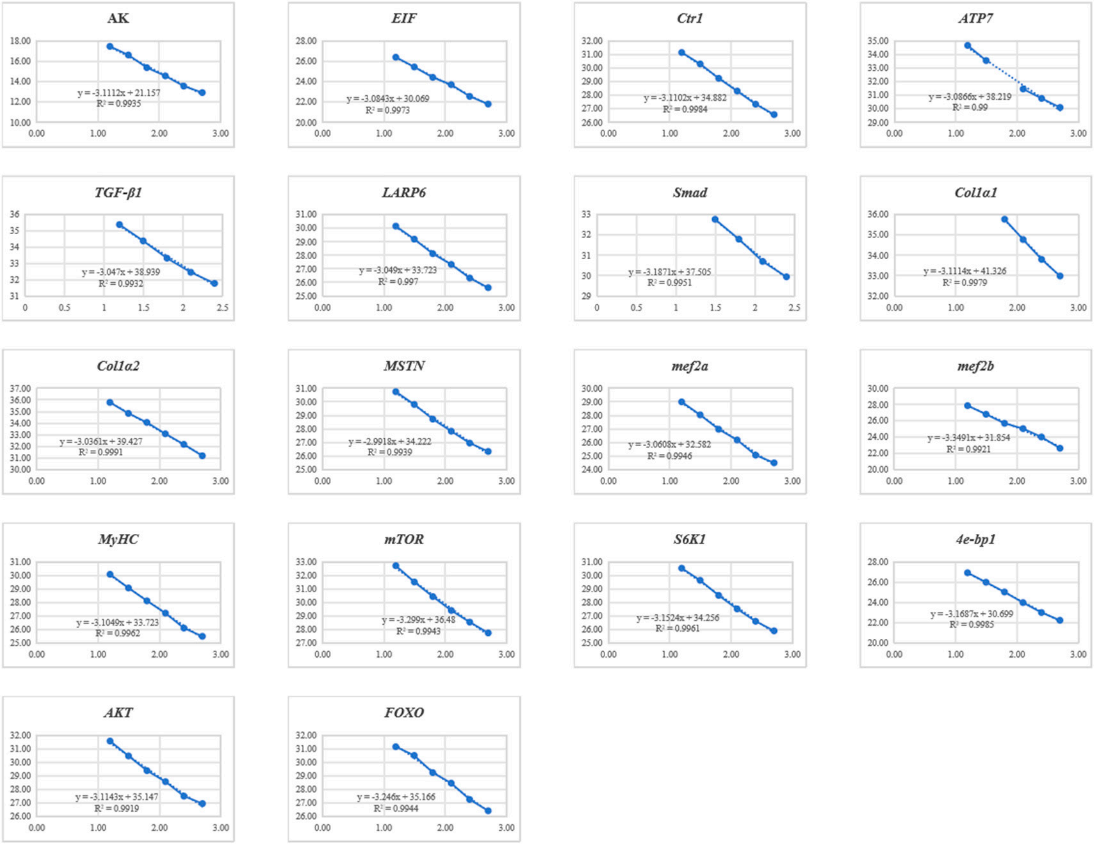

Figure S1. The reaction efficiency of different genes by qPCR analysis. The accuracy of real-time PCR assays was high ($R^2 > 0.99$) for all diluents based on linear regression analysis.

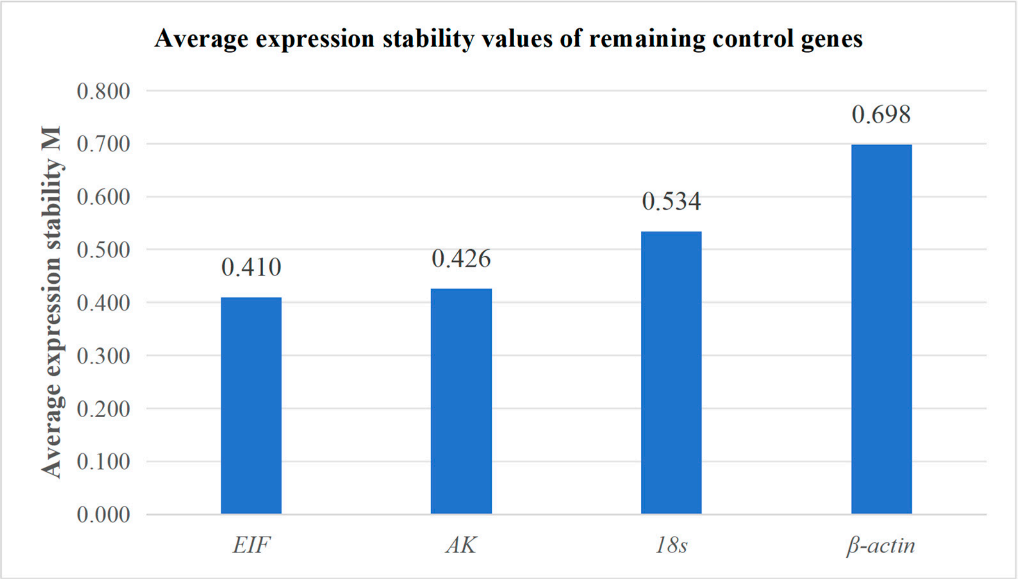

Figure S2. Ranking of candidate reference genes based on their expression stability measured by the geNorm algorithm.

Table S1. Approximate chemical composition of the whole crayfish body at the initial and final stages.

Items	initial	final				
		Cu1.48	Cu12.72	Cu27.71	Cu65.09	Cu121.34
Moisture(%)	71.52 ± 0.19	68.42 ± 0.15	68.52 ± 0.28	68.70 ± 0.21	68.59 ± 0.16	68.63 ± 0.28
Crude protein(%)	10.70 ± 0.13	10.06 ± 0.32	10.14 ± 0.10	11.11 ± 0.11	10.84 ± 0.11	10.12 ± 0.10
Crude lipid(%)	4.03 ± 0.08	4.27 ± 0.07	4.16 ± 0.07	4.08 ± 0.12	4.21 ± 0.06	4.29 ± 0.04
Ash(%)	10.10 ± 0.06	8.47 ± 0.10	8.47 ± 0.06	8.34 ± 0.07	8.45 ± 0.04	8.46 ± 0.03
